# Functional characterization of 11 novel rhoptry proteins in the type I RH strain of *Toxoplasma gondii* using the CRISPR-Cas9 system

**DOI:** 10.1186/s13071-026-07387-0

**Published:** 2026-04-13

**Authors:** Hong-Yu Song, Hui Cao, Shi-Bo Huang, Hany M. Elsheikha, Zhi Zheng, Xin-Sheng Lu, Xing Tian, Xiao-Nan Zheng, Xing-Quan Zhu

**Affiliations:** 1 Shanxi Key Laboratory of Animal Disease Research, Prevention and Control, College of Veterinary Medicine, Shanxi Agricultural University, Taigu, Jinzhong, 030801 Shanxi People’s Republic of China; 2 Department of Human Anatomy, Changzhi Medical College, Changzhi, 046000 Shanxi People’s Republic of China; 3 Faculty of Medicine and Health Sciences, School of Veterinary Medicine and Science, University of Nottingham, Sutton Bonington Campus, Loughborough, LE12 5RD UK

**Keywords:** *Toxoplasmagondii*, Rhoptry proteins, Clustered regularly interspaced short palindromic repeats, Clustered regularly interspaced short palindromic repeats-associated protein 9, Virulence, Transcriptomics

## Abstract

**Background:**

Rhoptry proteins (ROPs) are secreted effectors that play important roles in the virulence of *Toxoplasma gondii* by facilitating host cell invasion and immune modulation. Although many ROPs have been predicted, their specific functions remain largely unexplored. This study investigates the roles of 11 previously uncharacterized ROPs in *T. gondii* biology, with a focus on their contributions to virulence.

**Methods:**

Clustered regularly interspaced short palindromic repeats (CRISPR)–CRISPR-associated protein 9 (Cas9)–mediated genome editing was employed to generate epitope-tagged and knockout mutants for each candidate ROP in the* T. gondii* RHΔ*ku80* strain. Subcellular localization was determined via immunofluorescence microscopy in both tachyzoite and bradyzoite stages. In vitro assays assessed parasite invasion, replication, egress, and plaque formation. In vivo virulence was evaluated in mouse infection models. To explore molecular mechanisms underlying virulence attenuation, we performed transcriptomic profiling of RHΔ*rop64* and RHΔ*rop65* knockout strains.

**Results:**

All 11 candidate ROPs exhibited rhoptry localization in both tachyzoite and bradyzoite stages. Despite no apparent in vitro growth defects, deletion of ROP64 and ROP65 led to significant attenuation of virulence in mice, with ROP64 showing the most pronounced effect. Transcriptome analysis revealed downregulation of key immune-modulatory genes, including *ROP5*, *ROP39*, *TgIST*, and *PLP1*. In addition, RHΔ*rop64* exhibited broader suppression of ROPs than RHΔ*rop65*, suggesting it has a more pronounced role in immune modulation.

**Conclusions:**

ROP64 and ROP65 are critical to *T. gondii* virulence, likely through modulation of the parasite's immune-evasive machinery. Their regulatory influence on effector expression underscores their importance in host adaptation. Importantly, the RHΔ*rop64* mutant displays characteristics of an attenuated strain with potential for vaccine development against toxoplasmosis.

**Graphical Abstract:**

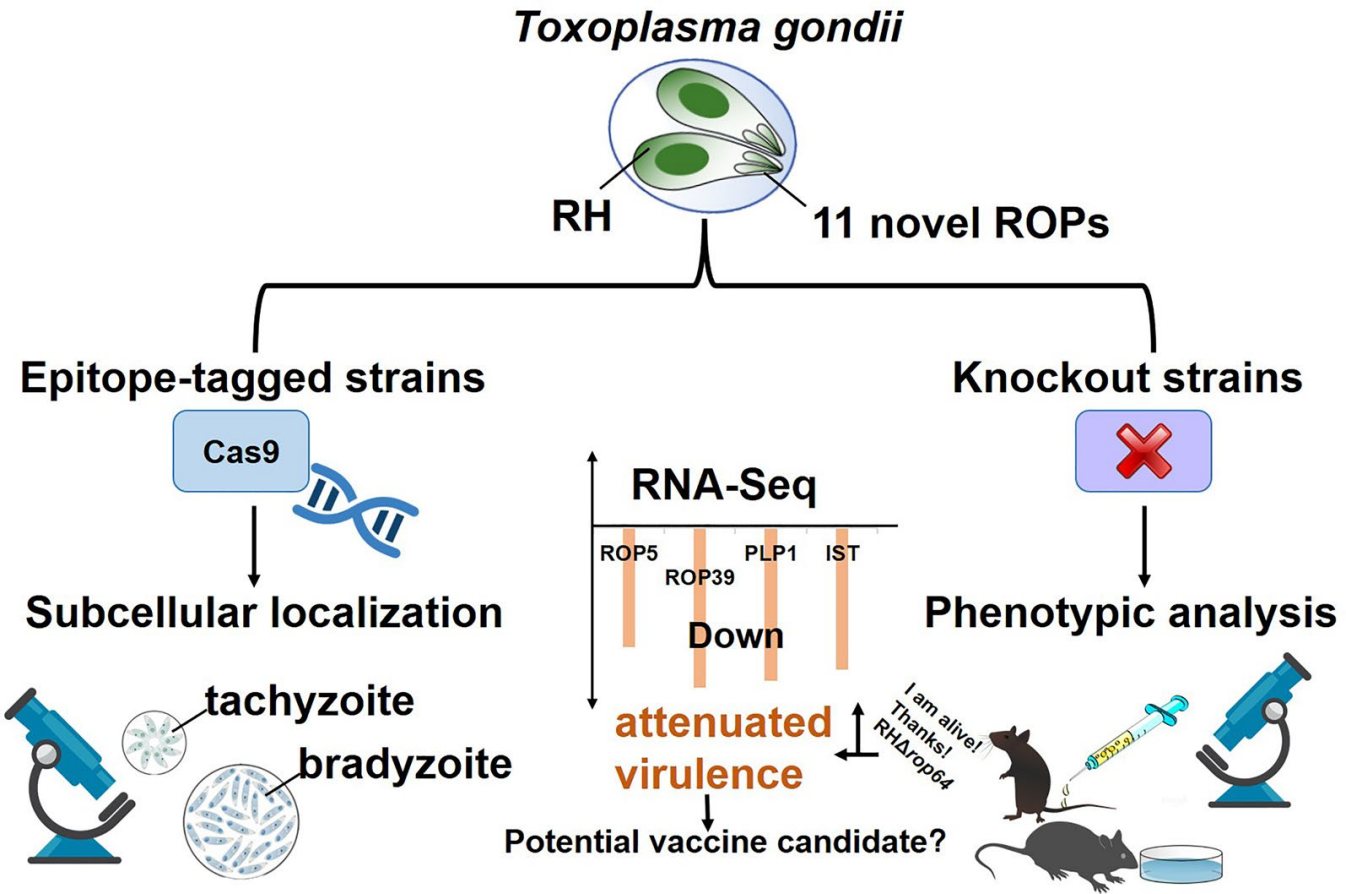

**Supplementary Information:**

The online version contains supplementary material available at 10.1186/s13071-026-07387-0.

## Background

*Toxoplasma gondii*, an apicomplexan with a global distribution, is capable of infecting nearly all species of warm-blooded animals, including humans [1–3]. Serological studies have estimated that nearly one-third of the global human population has been exposed to *T. gondii*, though prevalence rates vary widely by geography, socioeconomic status, and dietary practices [4, 5]. In immunocompetent individuals, infection is typically asymptomatic or leads to a latent chronic state. However, in immunocompromised patients and during pregnancy, infection can cause life-threatening complications, including encephalitis and congenital defects [6].

This parasite's ability to establish and maintain infection relies on the coordinated secretion of effector proteins from specialized secretory organelles, including micronemes, rhoptries, and dense granules. Each organelle releases distinct classes of proteins that mediate critical processes, including host cell invasion, formation of the parasitophorous vacuole (PV), nutrient acquisition, and immune evasion [6, 7]. Rhoptry proteins (ROPs) are secreted at the early stages of host cell invasion and are delivered either to the parasitophorous vacuole membrane (PVM) or directly into the host cell cytoplasm and nucleus, where they contribute to PV formation and modulate host signaling pathways and innate immune responses [8].

Several ROPs, especially those with kinase domains, have been well characterized as key virulence factors. For example, ROP16 activates host signal transducer and activator of transcription (STAT) signaling, while the ROP5–ROP17–ROP18 complex on the PVM inhibits immunity-related GTPases (IRGs), enabling parasite survival within macrophages [8–17]. ROP2 contributes to PVM–host interactions and cyst development, with its deletion impairing chronic infection in mouse models [18, 19]. These findings underscore the importance of ROPs not only in acute infection but also in chronic persistence.

Large-scale proteomic surveys have considerably expanded the catalog of predicted ROPs in *T. gondii*, yet the functions of many remain uncharacterized. In a pioneering study, Camejo et al. [20] identified new rhoptry residents by combined cell cycle–guided transcriptomics and epitope tagging, including ROP47 and ROP48, demonstrating that expression timing can predict organelle-specific localization. More recently, Barylyuk et al. [21] employed hyperplexed localization of organelle proteins by isotope tagging (hyperLOPIT) to generate a spatial proteomic atlas of *T. gondii*, assigning thousands of proteins to discrete subcellular compartments, including the rhoptries, with high confidence. Eleven candidate ROPs analyzed in the present study were previously flagged as rhoptry-localized proteins in these datasets. However, their functions have not been experimentally tested, leaving a critical gap between protein discovery and functional insight. Our study addresses this gap directly by combining precise genome editing with phenotypic and transcriptomic analyses to evaluate their roles in parasite virulence and host interaction.

Given the central role of ROPs in parasite survival and immune evasion, they are also considered promising targets for therapeutic intervention and vaccine development. In this study, we selected 11 candidate ROP genes from the *T. gondii* hyperLOPIT dataset (Table 1) that were predicted to localize to the rhoptry compartment. Using clustered regularly interspaced short palindromic repeats (CRISPR)–CRISPR-associated protein 9 (Cas9) gene editing in the *T. gondii* RHΔ*ku80* (hereafter “RH”) strain, we generated both gene knockouts and epitope-tagged lines. Through a combination of subcellular localization studies, in vitro functional assays, in vivo virulence testing, and transcriptomic profiling, we aimed to uncover previously unknown roles for these candidate ROPs in *T. gondii* biology and pathogenesis.

**Table 1:** Bioinformatic characteristics of 11 novel rhoptry proteins (*ROP*s) in *Toxoplasma gondii*

Gene identifier (ID)	New designation	Product description	Exons	Phenotype scores	Predicted location	HyperLOPIT localization scores	Molecular weight (kDa)	TMHMM^a^	Predicted signal peptide^b^
TGME49_312150	ROP56	Hypothetical protein	6	-0.23	Rhoptries 2	1	60.547	Yes	Yes
TGME49_246178	ROP57	Hypothetical protein	4	0.2	Rhoptries 2	1	52.134	Yes	No
TGME49_254070	ROP58	Hypothetical protein	1	-1.03	Rhoptries 2	1	25.957	Yes	No
TGME49_254880	ROP59	Alpha-galactosidase	11	-1.37	Rhoptries 2	1	81.795	Yes	Yes
TGME49_264600	ROP60	Hypothetical protein	2	0.95	Rhoptries 2	1	23.871	Yes	No
TGME49_270200	ROP61	Transporter, major facilitator family protein	2	0.65	Rhoptries 2	1	71.501	Yes	No
TGME49_271270	ROP62	Hypothetical protein	10	-0.55	Rhoptries 2	1	93.101	Yes	Yes
TGME49_273860	ROP63	Hypothetical protein	1	0.95	Rhoptries 1	1	37.907	No	Yes
TGME49_279420	ROP64	Hypothetical protein	1	-1.96	Rhoptries 1	1	144.942	No	Yes
TGME49_305270	ROP65	Hypothetical protein	6	-1.08	Rhoptries 1	1	82.315	No	Yes
TGME49_306895	ROP66	Hypothetical protein	1	1.36	Rhoptries 2	1	18.252	Yes	No

*HyperLOPIT* Hyperplexed localization of organelle proteins by isotope tagging

^a^Transmembrane helices were predicted using TMHMM version 2.0 (available at https://services.healthtech.dtu.dk/services/TMHMM-2.0/)

^b^Signal peptide predictions were conducted using SignalP version 6.0 (available at https://services.healthtech.dtu.dk/services/SignalP-6.0/)

## Methods

### Bioinformatics analysis

Eleven candidate genes encoding putative ROPs were selected from the *T. gondii* hyperLOPIT spatial proteomics dataset, integrated within the ToxoDB database [21]. This dataset assigns proteins to specific subcellular compartments with high confidence using mass spectrometry–based spatial proteomics. We focused on proteins predicted to localize to the rhoptry, which formed the basis for candidate selection in this study. The corresponding gene identifiers and related metadata are provided in Table 1. Following current community conventions, the selected genes were assigned streamlined designations ranging from ROP56 to ROP66, as no ROP numbers beyond ROP55 were previously in use (confirmed via EuPathDB/ToxoDB) [22]. These designations are used consistently throughout the manuscript and summarized in Table 1. Additional bioinformatic features, including predicted molecular weight, number of exons, CRISPR phenotype scores, predicted location, and hyperLOPIT localization confidence, were retrieved from ToxoDB. To further characterize candidate proteins, we predicted transmembrane domains using TMHMM version 2.0 (https://services.healthtech.dtu.dk/services/TMHMM-2.0/) and signal peptides using SignalP version 6.0 (https://services.healthtech.dtu.dk/services/SignalP-6.0/).

### Cell culture and parasite maintenance

Human foreskin fibroblast (HFF) cells (SCRC-1041; American Type Culture Collection, USA) were cultured, as previously described, in Dulbecco’s modified Eagle medium (Gibco, USA) supplemented with 100 μg/mL penicillin, 100 μg/mL streptomycin, 10 mM 4-(2-hydroxyethyl)-1-piperazineethanesulfonic acid (Solarbio, China), and 10% fetal bovine serum (Gibco) [23]. The RH strain, along with epitope-tagged and knockout mutants for the 11 candidate ROPs, were propagated on confluent HFF monolayers in T25 flasks (Thermo Fisher Scientific, USA). Parasites were maintained at 37 °C in a humidified atmosphere with 5% CO_2_ in Dulbecco’s modified Eagle medium containing 2% fetal bovine serum.

### Generation of epitope-tagged strains

**Fig. 1a, b:**
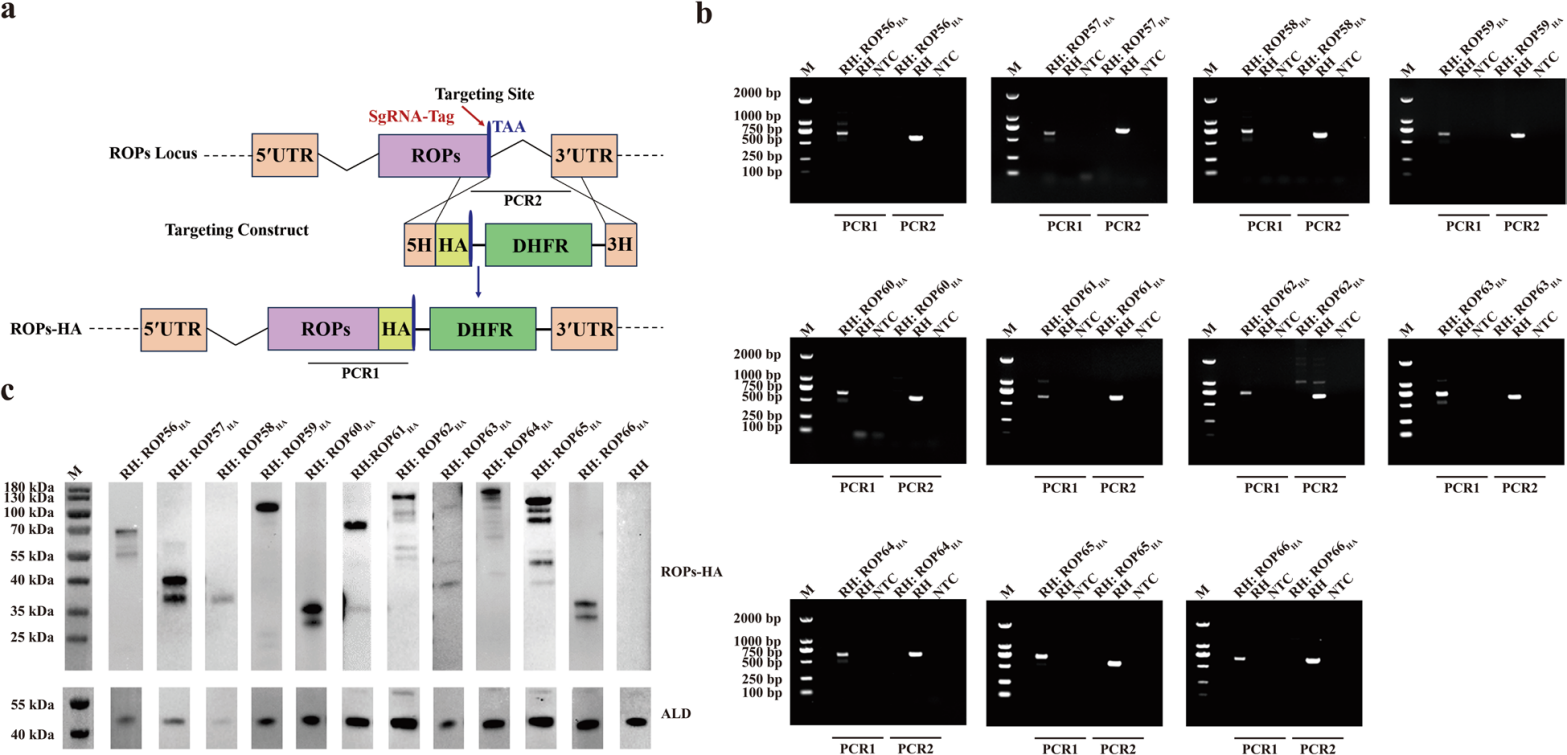
Generation of 11 novel rhoptry protein (*ROP*) epitope-tagged strains. **a** Schematic representation of the clustered regularly interspaced short palindromic repeats (CRISPR)–CRISPR-associated protein 9 (Cas9) strategy used to introduce C-terminal hemagglutinin (HA) tags into the 11 ROP genes. **b** Polymerase chain reaction (PCR) confirmation of successful HA tag integration in each ROP-tagged strain. **c** Western blot analysis verifying expression of HA-tagged proteins in the corresponding strains.* NTC* No template control

As illustrated in Fig. 1a, single guide RNAs (sgRNAs) targeting the 3′ end of each ROP gene were designed using the E-CRISP tool (http://www.e-crisp.org/). The pSAG1-Cas9-SgUPRT plasmid served as a backbone to generate gene-specific sgRNA-Tag constructs by replacing the SgUPRT region via polymerase chain reaction (PCR) amplification with Phanta® Max Super-Fidelity DNA Polymerase (Vazyme, China). The PCR products were digested with *Dpn*I (New England Biolabs, USA), circularized using KLD Enzyme Mix (New England Biolabs), and transformed into *Escherichia coli* Trans-T1 chemically competent cells (TransGen Biotech, China). Positive clones were confirmed by PCR and sequencing, followed by plasmid extraction with the TIANprep Mini Plasmid Kit (TIANGEN, China).

Homology-directed repair templates were generated by amplifying hemagglutinin (HA) epitope fragments, including 42-base pair (bp) flanking homology arms, and a dihydrofolate reductase (DHFR) resistance cassette from the p6 × HA-LIC-DHFR plasmid. For each gene, 35 μL of the gene-specific CRISPR-Cas9 plasmid and 30 μL of the corresponding homology-directed repair template were co-electroporated into RHΔ*ku80* tachyzoites. After 24 h, selection was applied using 3 μM pyrimethamine. Clonal populations were isolated by limiting dilution in 96-well plates and screened by PCR and sequencing [24]. Successfully tagged clones were then used for western blot and immunofluorescence assays (IFAs). Detailed sgRNA sequences and primer information are provided in Additional file 1: Table S1.

### Construction of CRISPR-Cas9 knockout strains

Following the strategy outlined in Fig. 4a, CRISPR-Cas9 knockout plasmids and homologous recombination templates containing the DHFR resistance cassette were constructed for each of the 11 novel ROP genes [25]. Gene-specific sgRNAs were designed and cloned into the pSAG1-Cas9-SgUPRT backbone via PCR amplification. After digestion, ligation, transformation into *E. coli*, and sequence verification, the plasmids were amplified and purified for subsequent use. Homologous recombination templates were generated by PCR amplification of 5′ and 3′ homology arms from RH genomic DNA. The DHFR resistance cassette was amplified from the pUPRT-DHFR-D plasmid, and the backbone fragment was obtained from pUC19. These fragments were seamlessly assembled using a cloning kit (TransGen Biotech) and transformed into competent *E. coli* cells. Verified plasmids served as templates to amplify the homologous DHFR fragments. For gene disruption, the CRISPR-Cas9 plasmid and corresponding homologous DHFR fragment were co-electroporated into RHΔ*ku80* tachyzoites. Following electroporation, parasites were subjected to pyrimethamine selection, and clonal populations were isolated. Successful gene knockouts were confirmed by PCR. All sgRNA sequences and primer details are provided in Additional file 2: Table S2.

### Western blotting analysis

Extracellular tachyzoites from epitope-tagged strains were harvested from the T25 flasks by centrifugation at 2000 r.p.m. for 10 min and washed with ice-cold phosphate-buffered saline (PBS) (Solarbio). Parasites were lysed on ice for 1 h in 150 μL radioimmunoprecipitation assay buffer (Thermo Fisher Scientific) supplemented with protease inhibitor and ethylenediaminetetraacetic acid. Lysates were resolved by SDS-PAGE and transferred onto polyvinylidene difluoride membranes. Membranes were incubated overnight with rabbit anti-HA (1:1000) (Cell Signaling Technology, USA) and rabbit anti-aldolase (1:500) primary antibodies, followed by goat anti-rabbit immunoglobulin G (IgG) secondary antibody (1:5000) (Biodragon, China). Protein expression of candidate ROPs was detected using enhanced chemiluminescence reagents (Thermo Fisher Scientific).

### Indirect IFA

The subcellular localization of the 11 novel ROPs was examined in both tachyzoite and bradyzoite stages of *T. gondii* using an indirect IFA. For tachyzoites, parasites were seeded onto HFF-coated confocal dishes and allowed to invade for 4 h. After washing with PBS, cultures were incubated for an additional 24 h. Cells and parasites were then fixed with 4% paraformaldehyde, permeabilized with 0.2% Triton X-100, and blocked with 5% bovine serum albumin (BSA). Primary antibodies, mouse anti-HA (1:300) (Cell Signaling Technology) and rabbit anti-armadillo repeat only protein (ARO) (1:300), were diluted in 3% BSA and incubated overnight at 4 °C. Secondary antibodies, Alexa Fluor™ Plus 594-conjugated goat anti-mouse IgG (H + L) and Alexa Fluor™ Plus 488-conjugated goat anti-rabbit IgG (H + L) (both 1:500) (Thermo Fisher Scientific) were applied for 1 h in the dark at 37 °C. Nuclei were stained with 4′,6-diamidino-2-phenylindole (DAPI; 1:500) for 20 min. Samples were visualized using a Nikon ECLIPSE Ti2 inverted fluorescence microscope.

For bradyzoite induction, cultures were switched to pH-8.2 medium under CO_2_-free conditions 4 h post-invasion. The medium was refreshed after 24 h, and incubation continued for another 24 h. Samples were fixed and stained as above, using mouse anti-HA and rabbit anti-ARO antibodies. Secondary antibodies included Alexa Fluor™ Plus 594-conjugated goat anti-mouse IgG (1:500), Alexa Fluor™ Plus 647-conjugated goat anti-rabbit IgG (1:500) (Thermo Fisher Scientific), and fluorescein isothiocyanate-conjugated *Dolichos biflorus* agglutinin (1:500) (Vectorlabs, USA) to label the cyst wall. Nuclei were counterstained with DAPI (1:500), and samples were examined by fluorescence microscopy.

### Invasion assay

Freshly egressed tachyzoites from the 11 confirmed RHΔ*rop* knockout strains and the parental RH strain were harvested, diluted, and 2 × 10^6^ tachyzoites were added to HFF-coated confocal dishes. After incubating for 1 h at 37 °C, the culture medium was removed, and cells were fixed with fixative solution (Solarbio) at room temperature for 30 min. Extracellular tachyzoites were labeled with mouse anti-SAG1 antibody (1:200) (Thermo Fisher Scientific) followed by Alexa Fluor™ Plus 594-conjugated goat anti-mouse IgG (H + L) secondary antibody (1:500). After permeabilization with 0.2% Triton X-100, all tachyzoites, both extracellular and intracellular, were stained with rabbit anti-IMC1 (1:500) and Alexa Fluor™ Plus 488-conjugated goat anti-rabbit IgG (H + L) (1:500). Fluorescence microscopy was used to image the samples. Invasion efficiency was quantified by counting approximately 200 tachyzoites across multiple random fields, differentiating extracellular (red-labeled) from total (green-labeled) parasites. The invasion rate was calculated using the formula: invasion (%) = [(green − red) / green] × 100% [26].

### Intracellular replication and egress assays

To evaluate intracellular replication and egress efficiency of the 11 RHΔ*rop* knockout strains, freshly egressed tachyzoites from each mutant and the RH strain were counted and diluted to 5 × 10^6^ tachyzoites/mL. For the replication assay, 100 μL of each suspension was added to HFF monolayers cultured in dishes. After 4 h of invasion, non-invaded tachyzoites were removed by washing with PBS, fresh medium was added, and cultures were incubated for an additional 30 h. The number of tachyzoites per PV was then counted microscopically, and the number of PVs containing 2, 4, 8, or 16 tachyzoites recorded. At least 100 PVs were analyzed per strain [27]. For the egress assay, cultures were incubated for 36 h. The spent medium was replaced with pre-warmed medium containing 3 μM A23187 to induce parasite egress, which was immediately monitored under the microscope. Once egress was observed, cells and parasites were fixed for 30 min at room temperature. The numbers of egressed and non-egressed PVs were counted, and the egress efficiency was calculated using the formula: egress (%) = (egressed PVs / total PVs) × 100%. At least 100 PVs were counted per strain [27].

### Plaque formation assay

To assess in vitro lytic growth, freshly egressed tachyzoites from each knockout strain and the RH control were diluted to 2 × 10^3^ tachyzoites/mL. Then, 100 μL of each suspension (equivalent to 200 tachyzoites per well) was inoculated onto confluent HFF monolayers in 12-well plates. Following 11 days of incubation, cells and parasites were fixed and stained with 0.5% crystal violet, then washed. Plaques were photographed and quantified using ImageJ software to measure plaque areas for statistical comparison [25, 28].

### Mouse infection assay

Specific pathogen-free female Kunming mice aged 6–8 weeks were obtained from Beijing SiPeiFu Biotechnology. The mice were randomly assigned to groups of six and housed together for 1 week prior to infection to minimize stress. During acclimatization, the mice had free access to food and water, and their bedding was changed every 2–3 days. Freshly egressed tachyzoites from RH and RHΔ*rop* strains were diluted to 5 × 10^2^ tachyzoites/mL, and each mouse was intraperitoneally injected with 200 μL of this suspension, which delivered 100 tachyzoites per mouse. Six mice were used per strain. Animals were monitored twice daily for clinical symptoms, and euthanasia was performed when humane endpoints were reached. Strains showing reduced virulence at this dose were further evaluated using a higher inoculum of 10^3^ tachyzoites per mouse. Infection was confirmed by culturing the same tachyzoite doses in HFF cells as previously described [29].

### Transcriptomic analysis of RHΔ*rop64* and RHΔ*rop65* strains

To investigate the molecular basis underlying the attenuated virulence observed in RHΔ*rop64* and RHΔ*rop65* strains, we performed RNA sequencing (RNA-seq) and differential gene expression analysis. Freshly egressed tachyzoites from RHΔ*rop64*, RHΔ*rop65*, and the parental RH strains were used to infect confluent HFF monolayers (three T75 flasks per strain, with three biological replicates each). When the majority of PVs contained 16 tachyzoites, cells and parasites were harvested, centrifuged at 1500 *g* for 10 min, washed 2 or 3 times with PBS, flash-frozen in liquid nitrogen, and stored at−80 °C until RNA extraction.

RNA isolation, library preparation, sequencing, and subsequent analyses were conducted by BGI-Shenzhen. Total RNA was extracted using TRIzol reagent, followed by removal of contaminating DNA with RNase-Free DNase (Thermo Fisher Scientific). RNA quality and integrity were assessed using NanoDrop spectrophotometry and an Agilent 2100 Bioanalyzer. Libraries were constructed following standard protocols including messenger RNA enrichment, fragmentation, complementary DNA synthesis, PCR amplification, and purification with AMPure XP beads. Sequencing was performed on the BGI-AEQ platform.

Raw sequencing was processed using SOAPnuke (version 1.6.5) to remove adapter sequence, low-quality reads, and reads containing more than 5% unknown bases. Clean reads were aligned to the *T. gondii* ME49 reference genome (https://toxodb.org/toxo/app) using HISAT2 (version 2.2.1). Gene expression levels were quantified as fragments per kilobase per million mapped fragments by using RSEM (version 1.3.1). Differential expression analysis was conducted with DESeq2 (version 1.40.2), applying thresholds of |log₂ fold change|≥ 1 and an adjusted* p*-value (*Q* -value) ≤ 0.05 to identify significantly transcribed genes.

### Reverse transcription quantitative real-time PCR

To validate the gene expression profiles obtained from RNA-seq, Reverse transcription quantitative real-time PCR (RT‑qPCR) was performed using a LightCycler 480 system (Roche, Basel, Switzerland). Twenty genes were selected for validation, including nine upregulated and 11 downregulated candidates. The RT‑qPCR protocol followed established methods as previously described [25]. Expression levels were normalized to the housekeeping gene *β-tubulin* (TGME49_266960) as an internal control. All reactions were performed in triplicate to ensure reproducibility and accuracy.

### Statistical analysis

All experiments were performed with a minimum of three independent biological replicates. Data analysis was conducted using GraphPad Prism 10. Statistical comparisons between groups were made using two-tailed unpaired Student's *t*-tests or one-way ANOVA, as appropriate. Differences were considered statistically significant at *P* ≤ 0.05.

## Results

### Successful construction of epitope-tagged strains

Bioinformatic analysis of the 11 novel putative ROPs is summarized in Table 1. To examine their subcellular localization, we generated epitope-tagged strains by inserting a 6 × HA tag at the C-terminus of each protein using CRISPR-Cas9 (Fig. 1a). All strains were validated by PCR and western blotting. As shown in Fig. 1b, PCR identification employed two primer sets (PCR1 and PCR2). PCR1 amplified specific fragments of approximately 500–750 bp in the tagged strains, confirming successful insertion of the 6 × HA tag at the 3′ end of each gene, while no product was detected in the parental RH strain. PCR2 yielded no product in tagged strains, reflecting replacement of the native 3′ untranslated region by the 6 × HA and DHFR sequences, which prevented amplification. In contrast, RH genomic DNA produced fragments of 500–600 bp, indicating unmodified ROP loci. Western blotting (Fig. 1c) confirmed expression of HA-tagged proteins in all 11 strains, with bands observed at predicted sizes consistent with ToxoDB annotations. Some strains exhibited additional bands, likely due to post-translational modifications. Collectively, these results demonstrate the successful generation of 11 epitope-tagged ROP strains.

### Subcellular localization of the 11 novel ROPs in *T. gondii*

**Fig. 2:**
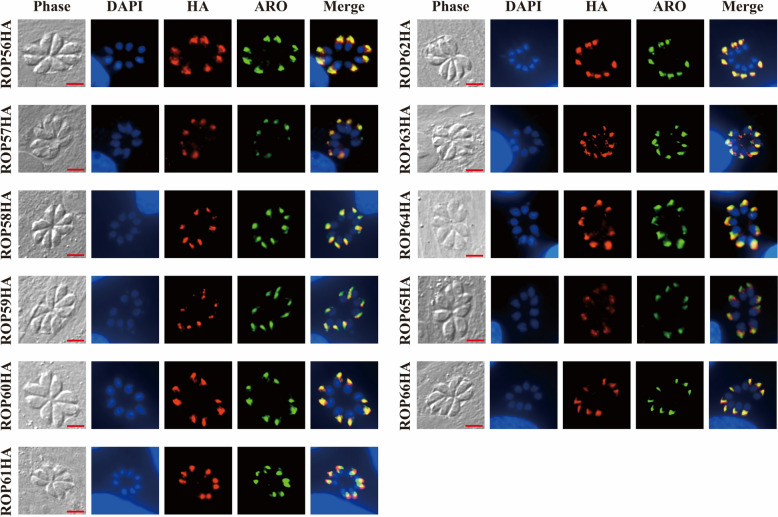
Subcellular localization of 11 novel ROPs in* Toxoplasma gondii* RHΔ*ku80* (RH) strain tachyzoites [co-staining with armadillo repeat only protein (ARO)]. HA-tagged ROPs are shown in red using an anti-HA antibody, rhoptry marker ARO is shown in green, and nuclei are shown in blue using 4′,6-diamidino-2-phenylindole (DAPI). Scale bar: 5 μm. All ROPs display localization consistent with rhoptry association in tachyzoites. For other abbreviations, see Fig. 1

IFA was performed to determine the localization of the 11 HA-tagged ROPs in tachyzoites. As shown in Fig. 2a, all 11 ROPs colocalized or partially colocalized with the rhoptry marker ARO, confirming their rhoptry localization.

**Fig. 3:**
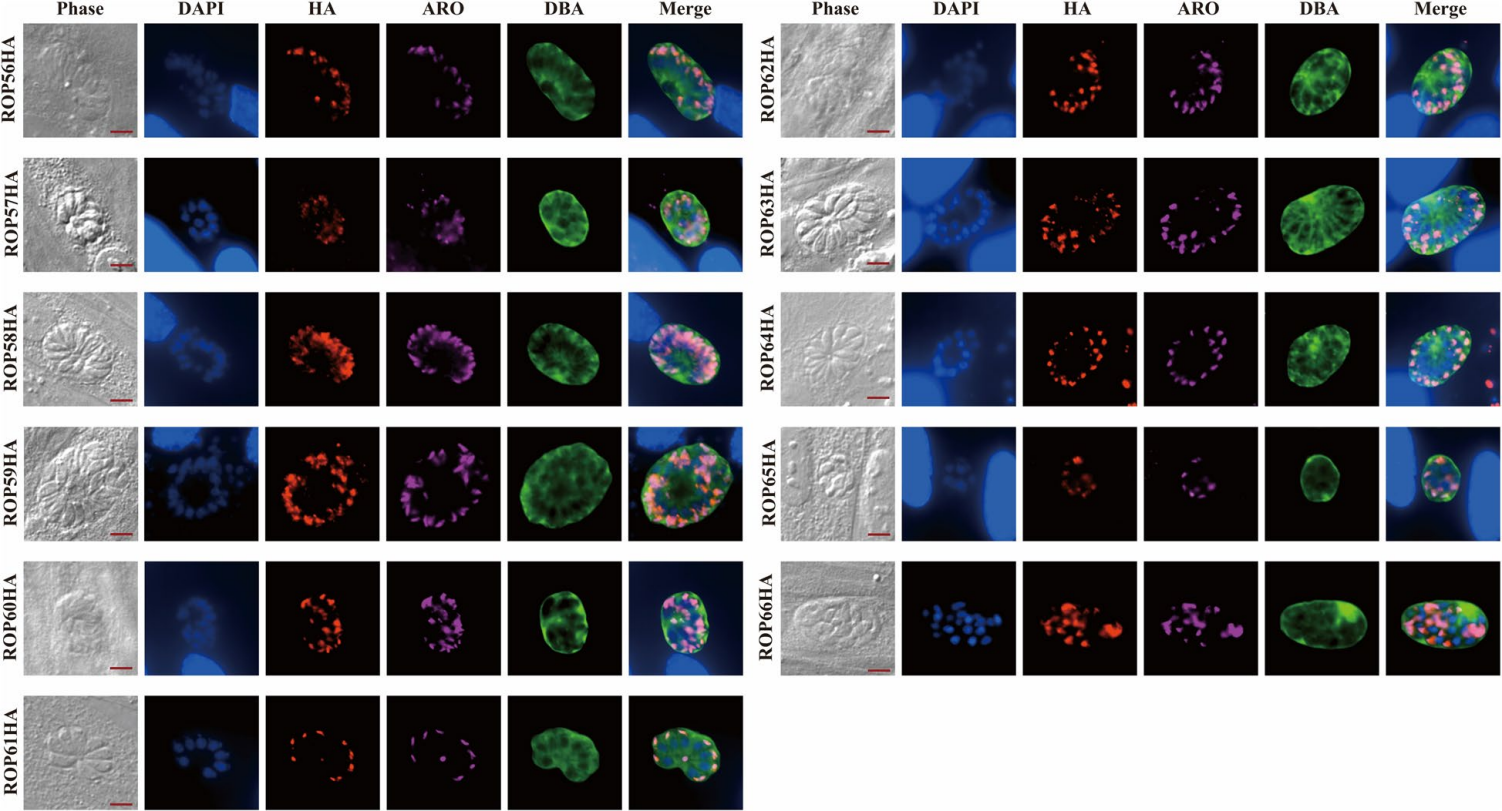
Subcellular localization of 11 novel ROPs in RH bradyzoites. HA-tagged ROPs are shown in red, the rhoptry marker ARO in magenta, cyst walls are stained green with fluorescein isothiocyanate-*Dolichos biflorus* agglutinin, and nuclei are shown in blue using DAPI. Scale bar: 5 μm. All ROPs demonstrate rhoptry localization in bradyzoites. For other abbreviations, see Figs. 1 and 2

*Toxoplasma gondii* tachyzoites can differentiate into bradyzoites within tissue cysts under stress conditions, such as elevated pH, low CO₂, or compromised host cell status. Prolonged induction periods typically yield larger cysts containing more bradyzoites. *Dolichos biflorus* agglutinin, which specifically binds N-acetylgalactosamine residues on the cyst wall, is commonly used to detect cyst or bradyzoite formation [30, 31]. To assess whether the localization patterns are stage-specific, bradyzoite differentiation was induced in vitro by allowing parasites to invade HFFs for 4 h, followed by alkaline stress induction (pH 8.2, CO_2_ free) for 48 h. As shown in Fig. 3a, IFA revealed that 11 ROPs retained rhoptry localization during the bradyzoite stage, consistent with their localization in tachyzoites. Collectively, the localization patterns of all 11 HA-tagged ROPs across both tachyzoites and bradyzoite stages support their classification as ROPs.

### Successful construction of knockout strains

**Fig. 4a, b:**
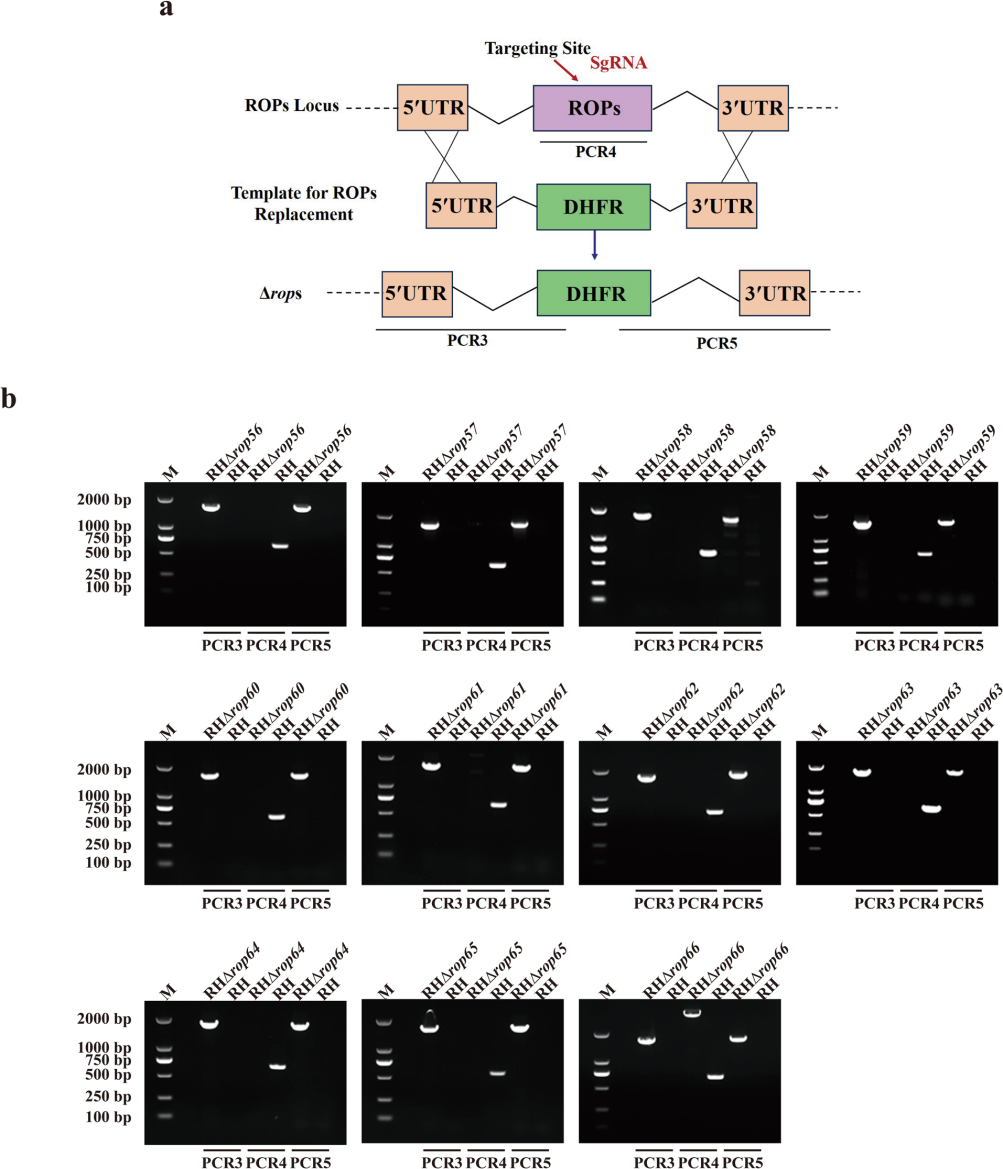
Generation of RHΔ*rop*s knockout strains. **a** Schematic overview of the CRISPR-Cas9 strategy used to generate 11 ROP knockout strains. **b** PCR verification confirming successful gene deletions in the RHΔ*rop*s strains. For other abbreviations, see Figs. 1 and 2

Knockout strains targeting the 11 novel ROP genes were generated using CRISPR-Cas9 technology (Fig. 4a). Gene deletions were confirmed by PCR analysis (Fig. 4b). In the RHΔ*rops* strains, the central regions of the target genes were replaced by DHFR fragments, resulting in the absence of PCR4 amplification, whereas the parental RH strain yielded a 400- to 600-bp band. Successful integration of the homologous DHFR cassette at the target loci was further verified by PCR3 and PCR5, which produced fragments of approximately 1000–1300 bp in the knockout strains but not in RH genomic DNA. These results confirm the successful generation of all 11 RHΔ*rops* knockout strains.

### The 11 novel ROPs are dispensable for the in vitro lytic cycle

**Fig. 5a–e:**
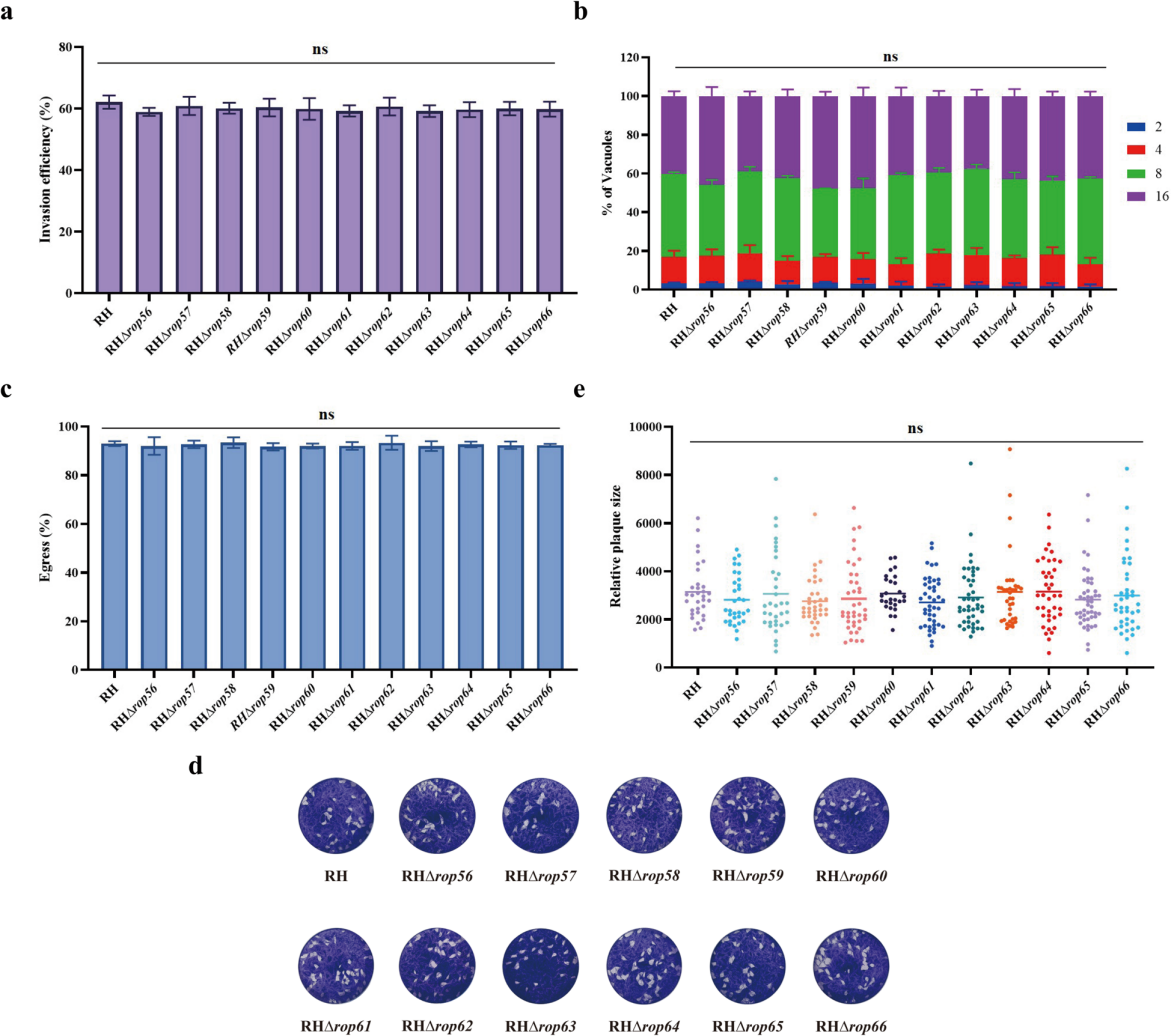
In vitro phenotypic analysis of RHΔ*rops* strains. **a** Invasion efficiency of knockout strains compared to the parental RH strain [not significant (ns), *P* > 0.05]. **b** Intracellular replication assessed by tachyzoite count per vacuole (ns, *P* > 0.05). **c** Egress efficiency following calcium ionophore induction (ns, *P* > 0.05). **d** Representative plaque images of the RH and RHΔ*rops* strains after 11 days of growth. **e** Quantitative measurement of plaque sizes, showing no significant differences (ns, *P* > 0.05)

To assess the impact of individual deletion of the 11 novel ROP genes on *T. gondii* growth in vitro, we conducted invasion, intracellular replication, egress, and plaque formation assays. Invasion efficiency, evaluated by IFA, showed no significant differences between the RHΔ*rops* knockout strains and the parental RH strain (Fig. 5a). Intracellular replication, measured by counting the number of tachyzoites per PV at 30 h post-infection, revealed a similar distribution of PVs containing 2, 4, 8, or 16 parasites across all strains, indicating no replication defects (Fig. 5b). Egress efficiency, induced by the calcium ionophore A23187, was comparable between knockout and parental strains, suggesting that these ROPs are dispensable for egress (Fig. 5c). Finally, plaque assays demonstrated no significant differences in plaque size between knockout and parental strains, confirming that deletion of these ROPs does not affect the parasite's lytic cycle in vitro (Fig. 5d, e).

### TGME49_279420 (ROP64) and TGME49_305270 (ROP65) are important for acute toxoplasmosis in mice

**Fig. 6a, b:**
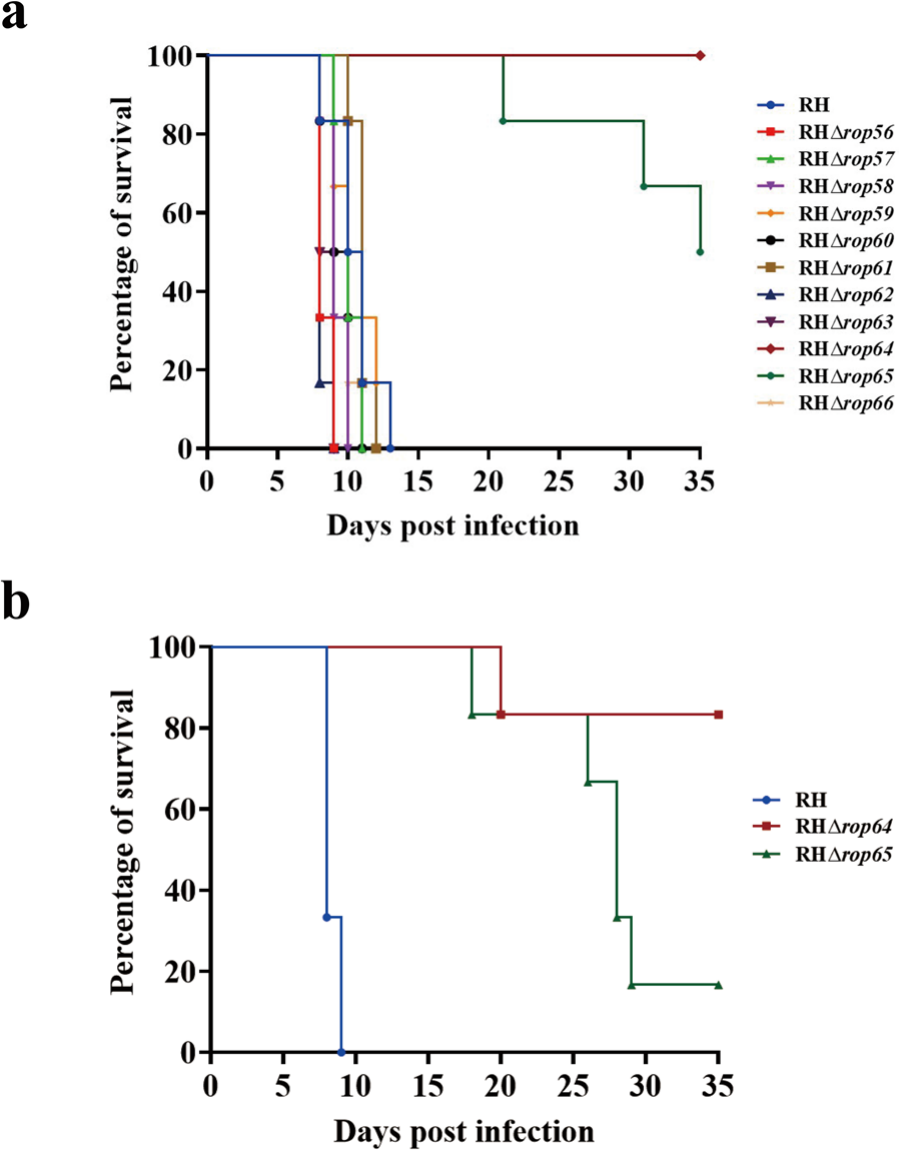
Virulence assessment of RHΔ*rop* strains in mice. **a** Survival curves of mice infected intraperitoneally with 100 tachyzoites of either the parental RH or various RHΔ*rop* strains. **b** Survival curves of mice infected with 1000 tachyzoites of RH, RHΔ*rop64*, or RHΔ*rop65* strains, highlighting differences in virulence

To evaluate the role of the novel ROPs in *T. gondii* virulence in vivo, mice were intraperitoneally injected with 100 tachyzoites of each strain. As shown in Fig. 6a, mice individually infected with nine of the 10 RHΔ*rops* strains (TGME49_312150 [ROP56], TGME49_246178 [ROP57], TGME49_254070 [ROP58], TGME49_254880 [ROP59], TGME49_264600 [ROP60], TGME49_270200 [ROP61], TGME49_271270 [ROP62], TGME49_273860 [ROP63], and TGME49_306895 [ROP66]) reached humane endpoints within 13 days post-infection, similar to those infected with the parental RH strain. In contrast, mice infected with RHΔ*rop65* (TGME49_305270) exhibited delayed mortality, with 50% survival at 35 days. Remarkably, all mice infected with RHΔ*rop64* (TGME49_279420) survived. Subsequent testing with a higher dose (10^3^ tachyzoites) confirmed the attenuated virulence of both RHΔ*rop65* and RHΔ*rop64* strains, with survival rates of 16.67% and 83.33%, respectively (Fig. 6b). These results demonstrate that ROP64 and ROP65 are significant contributors to *T. gondii* acute virulence.

### Deletion of *rop64* and *rop65* downregulates virulence-related effectors

**Fig. 7a–e:**
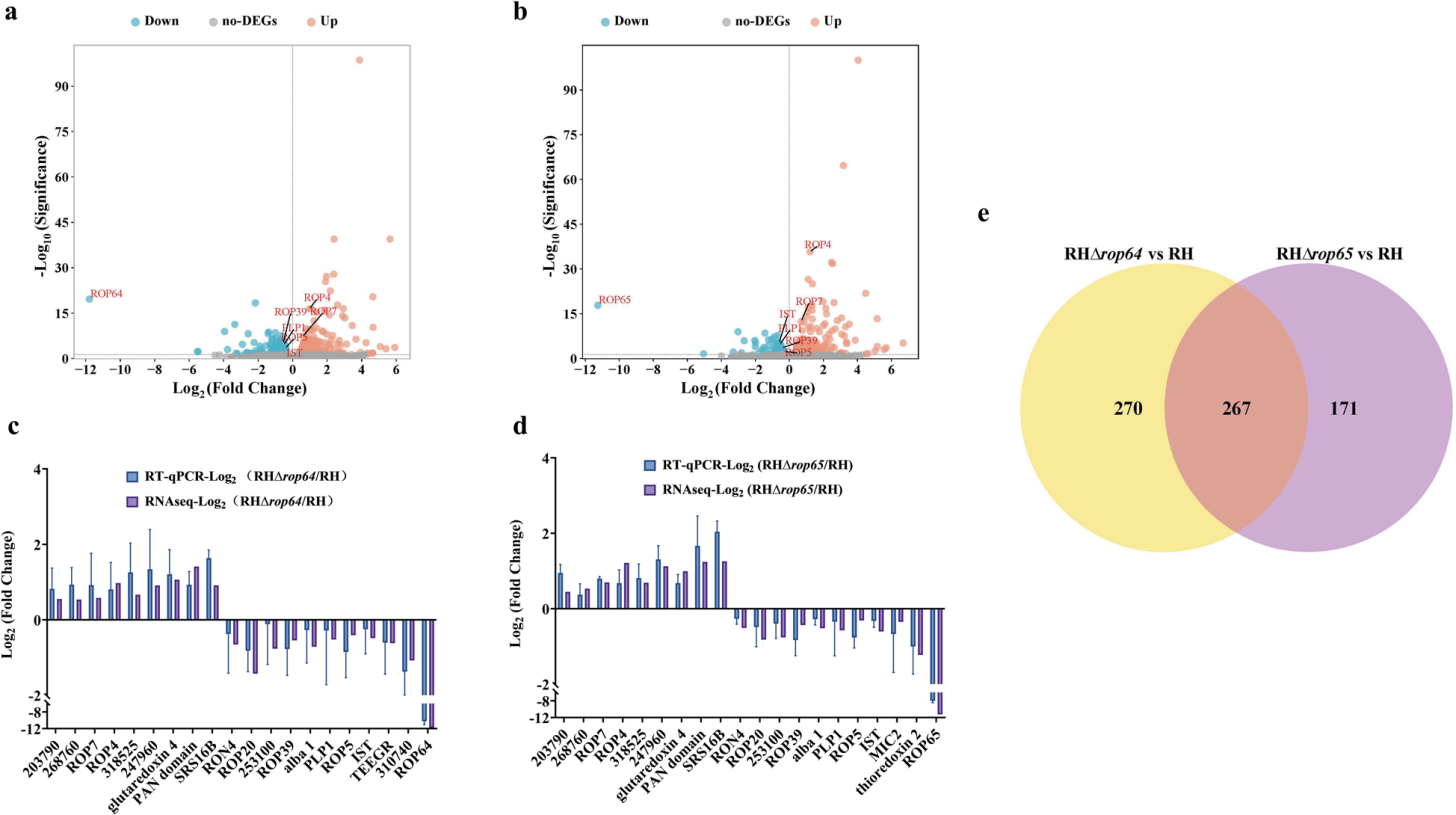
Transcriptomic analysis of RHΔ*rop64* and RHΔ*rop65* strains. **a** Volcano plot depicting differentially expressed genes (DEGs) in RHΔ*rop64* strain compared to the parental RH strain. **b** Volcano plot showing DEGs in RHΔ*rop65* strain versus RH strain. **c** Reverse transcription quantitative real-time PCR (RT‑qPCR) validation of selected DEGs in the RHΔ*rop64* strain. **d** RT-qPCR validation of selected DEGs in the RHΔ*rop65* strain. **e** Venn diagram illustrating the overlap of DEGs between RHΔ*rop64* and RHΔ*rop65* strains relative to the RH strain. For other abbreviations, see Figs. 1 and 2

Given the significantly reduced virulence of the RHΔ*rop64* and RHΔ*rop65* strains in mice, we conducted transcriptomic analysis to explore the underlying molecular mechanisms. As shown in Fig. 7a, b, the targeted genes themselves were not expressed in RHΔ*rop64* and RHΔ*rop65* strains. A total of 537 and 438 differentially expressed genes (DEGs) were identified in RHΔ*rop64* and RHΔ*rop65*, respectively. Specifically, RHΔ*rop64* showed 305 upregulated and 232 downregulated genes (Fig. 7a), while RHΔ*rop65* exhibited 313 upregulated and 125 downregulated genes (Fig. 7b). To validate the RNA-seq data, RT-qPCR was performed on 20 selected DEGs. As shown in Fig. 7c, d, the RT-qPCR results confirmed the expression trends observed in RNA-seq.

Subcellular localization predictions for these DEGs were analyzed (see Table 2) [21]. Given the critical role of ROPs in *T. gondii* virulence, we focused on DEGs encoding ROPs. In RHΔ*rop64*, seven rhoptry-localized genes were upregulated, whereas 29 were downregulated. In RHΔ*rop65*, eight ROPs were upregulated and nine were downregulated.

**Table 2:** Predicted subcellular localization of differentially expressed genes (DEGs) in the RHΔ*rop64* and RHΔ*rop65* strains

DEG sets	RHΔ*rop64* vs. RH		RHΔ*rop65* vs. RH	
Localization prediction	Upregulated	Downregulated	Upregulated	Downregulated
40S ribosome	22	0	18	0
60S ribosome	34	0	29	0
Apical 1	1	6	0	13
Apical 2	0	1	0	3
Apicoplast	4	3	9	0
Cytosol	24	16	38	5
Dense granules	23	15	19	10
Endomembrane vesicles	1	0	1	0
ER 1	2	9	4	2
ER 2	2	0	1	0
Golgi	0	4	2	0
IMC	4	13	1	11
Micronemes	7	2	6	4
Mitochondrion—membranes	4	0	5	0
Mitochondrion—soluble	4	0	2	0
Nucleolus	1	0	1	0
Nucleus—chromatin	19	18	14	11
Nucleus—non-chromatin	9	12	6	5
PM—integral	3	9	4	2
PM—peripheral 1	7	5	11	1
PM—peripheral 2	1	5	1	1
Rhoptries	7	29	8	9
Tubulin cytoskeleton	0	2	0	3
Not assessed	126	83	133	45
Total	305	232	313	125

*RH* RHΔ*ku80* strain; for other abbreviations, see Table 1

Comparative analysis of DEGs from both mutants (RHΔ*rop64* vs. RH and RHΔ*rop65* vs. RH) revealed 267 overlapping genes (Fig. 7e). Among rhoptry-localized DEGs, 13 ROPs were common to both strains (Table 3). Seven of these 13 ROPs were commonly upregulated and six were downregulated, among which were the well-established virulence factors ROP5 and ROP39. In addition to these shared ROPs, 23 ROPs were uniquely dysregulated in RHΔ*rop64*, all of which were downregulated; whereas only four ROPs were specific to RHΔ*rop65*, including one upregulated and three downregulated genes (Additional file 3: Table S3; Additional file 4: Table S4).

**Table 3:** Shared differentially expressed ROPs in the RHΔ*rop64* and RHΔ*rop65* knockout strains compared to the parental RH strain

Gene ID	Product description	Regulation
TGME49_203790	Hypothetical protein	Up
TGME49_268760	Hypothetical protein	Up
TGME49_295110	ROP7	Up
TGME49_295125	ROP4	Up
TGME49_318525	Hypothetical protein	Up
TGME49_247960	Hypothetical protein	Up
TGME49_270920	Rhoptry kinase family protein ROP32	Up
TGME49_229010	Rhoptry neck protein RON4	Down
TGME49_258230	Rhoptry kinase family protein ROP20	Down
TGME49_300100	Rhoptry neck protein RON2	Down
TGME49_308090	ROP5	Down
TGME49_253100	Hypothetical protein	Down
TGME49_262050	Rhoptry kinase family protein ROP39	Down

For abbreviations, see Table 1

Furthermore, several DEGs predicted to localize to dense granules and micronemes were identified in both mutants (Additional files 3, 4 and 5). Importantly, two key virulence-related effectors—*Tg*IST (TGME49_240060), a STAT1 transcription inhibitor, and perforin-like protein 1 (PLP1) (TGME49_204130), a perforin-like protein—were significantly downregulated in both RHΔ*rop64* and RHΔ*rop65* (Additional file 5: Table S5), suggesting a potential impairment in host immune modulation.

A summary table (Table 4) that collates the experimental outcomes of the present study provides an integrative overview of the functional characterization of the 11 candidate ROPs.

**Table 4:** Summary of experimental findings for the 11 candidate ROPs (ROP56–ROP66)

New designation	Tachyzoite localization^a^	Bradyzoite localization^a^	In vitro phenotype^b^	In vivo virulence phenotype^c^
ROP56	Rhoptry	Rhoptry	No change	No change
ROP57	Rhoptry	Rhoptry	No change	No change
ROP58	Rhoptry	Rhoptry	No change	No change
ROP59	Rhoptry	Rhoptry	No change	No change
ROP60	Rhoptry	Rhoptry	No change	No change
ROP61	Rhoptry	Rhoptry	No change	No change
ROP62	Rhoptry	Rhoptry	No change	No change
ROP63	Rhoptry	Rhoptry	No change	No change
ROP64	Rhoptry	Rhoptry	No change	Attenuated
ROP65	Rhoptry	Rhoptry	No change	Attenuated
ROP66	Rhoptry	Rhoptry	No change	No change

^a^Experimental localization confirmed by immunofluorescence assay using the rhoptry marker armadillo repeat only protein in both tachyzoites and bradyzoites

^b^In vitro phenotypic assays (invasion, replication, egress, and plaque formation) showed no significant differences compared to the parental RH strain for all knockout mutants

^c^In vivo virulence assessment via mouse survival assays demonstrated attenuated virulence only in RHΔ*rop64* and RHΔ*rop65* strains; all other knockouts exhibited no significant changes

## Discussion

This study closes a critical knowledge gap by functionally linking a set of previously uncharacterized ROPs to the virulence mechanisms of *T. gondii*, and it unveils promising new targets for therapeutic intervention and vaccine development. Leveraging CRISPR-Cas9–mediated epitope tagging and gene knockout approaches, we identified and characterized 11 novel ROPs in the highly virulent RH strain of *T. gondii*. Although recent advances, such as the HyperLOPIT technology employed by Barylyuk et al. [21], have mapped the subcellular localization of many putative ROPs, the biological functions of these proteins have largely remained unknown. Our study focused on 11 candidates bioinformatically predicted to localize specifically to the rhoptries, secretory organelles central to parasite invasion and host manipulation.

CRISPR-Cas9 technology has revolutionized the study of *T. gondii* cell biology, enabling precise manipulation and localization studies of key proteins such as GRA76 and PP2A [25, 32]. Applying this tool, we successfully generated epitope-tagged strains for each of these novel ROPs, confirming their expression and subcellular distribution. IFAs demonstrated that all 11 novel ROPs colocalize with the rhoptry marker ARO during the tachyzoite and bradyzoite stages, consistent with established ROP profiles [33]. This conserved localization across tachyzoite and bradyzoite stages supports their classification as bona fide ROPs, and suggests that their subcellular distribution may be maintained across multiple developmental stages of *T. gondii*. The consistent rhoptry localization of these proteins in both acute and chronic stages further implies that they may participate in core rhoptry-associated functions, potentially including host cell invasion, modulation of host signaling pathways, or the establishment and maintenance of chronic infection.

ROPs are well known for their multifaceted roles in host cell invasion, PVM remodeling, immune evasion, and intracellular survival [34]. Although individual deletion of these 11 ROPs did not impair classic in vitro phenotypes, including invasion, replication, egress, and plaque formation, two knockout strains, RHΔ*rop64* and RHΔ*rop65*, exhibited strikingly reduced virulence in vivo. RHΔ*rop64*, in particular, showed the most pronounced attenuation, highlighting these proteins as critical contributors to acute pathogenicity and positioning them as novel virulence factors.

Immune evasion lies at the heart of *T. gondii'*s success as a pathogen, enabling it to establish and persist within its host. A cornerstone of this evasion involves inactivating host IRGs and guanylate-binding proteins, key effectors induced by interferon-gamma that oligomerize on the PVM to mediate parasite clearance [35–37]. To counteract these defenses, *T. gondii* deploys polymorphic effector proteins, including a cadre of ROPs such as ROP5, ROP17, ROP18, and ROP39, which phosphorylate and neutralize IRGs, thereby preventing immune-mediated parasite destruction [35]. Our findings place ROP64 and ROP65 alongside these critical virulence factors, underscoring their potential role in subverting host immune responses and sustaining infection.

Given the pronounced impact of ROP64 and ROP65 deletion on virulence, we turned to transcriptomic profiling to uncover the molecular mechanisms underlying this attenuation. Strikingly, both RHΔ*rop64* and RHΔ*rop65* strains exhibited significant downregulation of several key rhoptry effectors, including the well-characterized virulence genes ROP5 and ROP39. ROP5 serves as a crucial allosteric inhibitor of IRGs, and its loss is known to completely abolish virulence in mouse models, while ROP39 directly binds to Irgb10, suppressing its dimerization and accumulation on the PVM, thereby promoting parasite survival within the host [17, 38, 39]. Although ROP39 alone modestly reduces virulence, combined deletion with ROP18 results in a profound attenuation [39]. The observed coordinated downregulation of these virulence effectors in our knockout strains likely contributes substantially to their impaired pathogenicity. Moreover, the broader suppression of ROP gene expression in RHΔ*rop64* may explain its more pronounced virulence defect compared to RHΔ*rop65*.

In addition to ROPs, both mutants demonstrated reduced expression of *Tg*IST and PLP1—two effectors critical for immune modulation and parasite egress, respectively. *Tg*IST is a potent inhibitor of STAT1-mediated transcription, dampening interferon signaling, while PLP1 controls PVM permeability during egress, facilitating parasite exit from host cells [40–43]. The concurrent downregulation of these effectors suggests a synergistic impairment of immune evasion and egress processes, which further weakens parasite fitness in vivo.

Interestingly, despite the downregulation of genes linked to invasion and egress, neither knockout strain exhibited detectable defects in these processes under standard in vitro conditions. This discrepancy likely reflects compensatory mechanisms or functional redundancy within the extensive ROP family, especially in the highly virulent RH strain, masking phenotypic consequences of single gene deletions. In support of this, genome-wide CRISPR-Cas9 fitness screens have revealed that relatively few rhoptry, microneme, or dense granule proteins exhibit strongly negative fitness scores in vitro, underscoring redundancy or substitutability of most secretory effectors in cultured cells [44]. Importantly, six of our 11 candidate ROPs showed negative fitness scores in these screens, with ROP64 exhibiting the most pronounced score, consistent with its marked in vivo attenuation.

These findings highlight the limitations of pooled in vitro screens and emphasize the necessity of focused single-gene knockout studies to reveal context-dependent functions—particularly those that emerge under host immune pressure or in vivo infection settings. Future investigations using immune-activated host cells may uncover subtle phenotypes that are masked under baseline culture conditions.

*Toxoplasma gondii* virulence is orchestrated by complex regulatory networks involving post-transcriptional modifications, secretory dynamics, and host immune interactions. While transcriptomics offers valuable clues, it alone cannot establish causal relationships. To fully elucidate the mechanistic roles of ROP64 and ROP65, future studies should integrate proteomics, secretion assays, host–pathogen interaction analyses, and in vivo immunological profiling. Additionally, host transcriptomic profiling under immune-stimulated conditions could shed light on how these ROPs modulate key signaling pathways, such as STAT1-mediated transcription.

Further validation in less virulent, cyst-forming strains such as ME49 or PRU, alongside functional complementation experiments, is essential to definitively confirm the direct contribution of ROP64 and ROP65 to virulence in chronic infection, given the RH strain's inherent inability to establish tissue cysts. Finally, while the RHΔ*rop64* strain emerges as a promising attenuated candidate, its potential as a vaccine strain warrants testing.

## Conclusions

We functionally characterized 11 novel ROPs in *T. gondii* and demonstrated that most are dispensable for in vitro growth. However, ROP64 and ROP65 contribute significantly to virulence in vivo, likely by regulating immune-related effectors. Transcriptomic analyses revealed downregulation of known virulence genes, including ROP5, ROP39, *Tg*IST, and PLP1, especially in the RHΔ*rop64* strain. Notably, all characterized ROPs exhibited conserved rhoptry localization in both tachyzoite and bradyzoite stages, supporting their classification as bona fide ROPs. Despite this conserved localization, differential expression patterns suggest that certain ROPs may play context-dependent roles during acute versus chronic infection. This work expands our understanding of ROP function, highlights potential immune evasion mechanisms, and identifies ROP64 as a promising target for vaccine development pending its further evaluation in cyst-forming strains and chronic infection models.

## Supplementary Information

Additional file 1. Table S1. Primers used for constructing the epitope-tagged strains.

Additional file 2. Table S2. Primers employed for generating gene knockout strains.

Additional file 3. Table S3. Differentially expressed ROP, GRA, and MIC genes uniquely identified in the RHΔ*rop64* strain.

Additional file 4. Table S4. Differentially expressed ROP, GRA, and MIC genes uniquely identified in the RHΔ*rop65* strain.

Additional file 5. Table S5. Shared differentially expressed GRA and MIC genes in both RHΔ*rop64* and RHΔ*rop65* knockout strains.

## Data Availability

The RNA-seq datasets generated and analyzed in this study have been deposited in the National Center for Biotechnology Information Sequence Read Archive under the BioProject accession no. PRJNA1309712. Other datasets supporting the findings of this article are included within the paper and its supplementary materials.

## Abbreviations

ARO: Armadillo repeat only protein
Cas9: Clustered regularly interspaced short palindromic repeats-associated protein 9
CRISPR: Clustered regularly interspaced short palindromic repeats
DAPI: 4′,6-Diamidino-2-phenylindole
DEG: Differentially expressed gene
DHFR: Dihydrofolate reductase
HA: Hemagglutinin
HFF: Human foreskin fibroblast
HyperLOPIT: Hyperplexed localization of organelle proteins by isotope tagging
IFA: Immunofluorescence assay
IgG: Immunoglobulin G
IRG: Immunity-related GTPase
PBS: Phosphate-buffered saline
PCR: Polymerase chain reaction
PLP1: Perforin-like protein 1
PV: Parasitophorous vacuole
PVM: Parasitophorous vacuole membrane
RT-qPCR: Reverse transcription quantitative real‑time polymerase chain reaction
RNA-seq: RNA sequencing
ROP: Rhoptry protein
sgRNA: Single guide RNA
STAT1: Signal transducer and activator of transcription 1
*Tg*IST: *Toxoplasma gondii* inhibitor of signal transducer and activator of transcription 1-dependent transcription
