# Supplementary material for: Functional characterization of 11 novel rhoptry proteins in the type I RH strain of Toxoplasma gondii using the CRISPR-Cas9 system
Source: Parasit Vectors. 2026 Apr 13;19:221. doi: 10.1186/s13071-026-07387-0 (PMC13185275; doi:10.1186/s13071-026-07387-0)
Supplement: Supplementary file 1 — Additional file 1. Table S1. Primers used for constructing the epitope-tagged strains. [file 13071_2026_7387_MOESM1_ESM.pdf]

Additional file 1: Table S1 Primers used for constructing the epitope-tagged strains.

Primer	Sequence (5′→3′)	Use
sgRNA-Tag-Rv	AACTTGACATCCCCATTTAC	Construct the CRISPR plasmid for tagging ROP genes with 6×HA (universal primer)
PCR1-Flag-Rv	ATTATACCCGTGTGTTACG	Detect the insert 6×HA fragment in PCR1 (universal primer)
sgRNA-TGME49_312150-Tag	CGATCAGACTTTAGTAGCTC	Designed SgRNA-Tag sequence for TGME49_312150
sgRNA-TGME49_312150-Tag-Fw	CGATCAGACTTTAGTAGCTCGTTTTAGAGCTAGAAATAGC	Construct the CRISPR plasmid for tagging TGME49_312150 with 6×HA
TGME49_312150-HR-Fw	GTCGTTGTCACATCAGACTGTGTCTCCGGAGCTACTAAAGTCGCTAGCAAGGGCTCGGG	Amplify 6HA-DHFR fragment with the homologous fragment of *TGME49_312150*
TGME49_312150-HR-Rv	TTGCGAAGCTAAAAAACGATCAGACTTTAGTAGCTCCGGAGAATACGACTCACTATAGG	Amplify 6HA-DHFR fragment with the homologous fragment of *TGME49_312150*
PCR1/2-TGME49_312150-Fw	GTGACATGCAGAGCAGCAAG	Detect the replacement of C-terminal TGME49_312150 by 6×HA fragment in PCR2
PCR2-TGME49_312150-Rv	GAAGTGCAAGCTGCGACAC	Detect the replacement of C-terminal TGME49_312150 by 6×HA fragment in PCR2
sgRNA-TGME49_246178-Tag	CTATTCAAGAGTGCACAAAG	Designed SgRNA-Tag sequence for TGME49_246178
sgRNA-TGME49_246178-Tag-Fw	CTATTCAAGAGTGCACAAAGGTTTTAGAGCTAGAAATAGC	Construct the CRISPR plasmid for tagging TGME49_246178 with 6×HA
TGME49_246178-HR-Fw	GACGCTTGTCAAGAACGCCATCGCCTCCTCTTTGTGCACTCTGCTAGCAAGGGCTCGGG	Amplify 6HA-DHFR fragment with the homologous fragment of *TGME49_246178*
TGME49_246178-HR-Rv	CACACAAACTATACACAGTGACTGACTATTCAAGAGTGCACAATACGACTCACTATAGG	Amplify 6HA-DHFR fragment with the homologous fragment of *TGME49_246178*
PCR1/2-TGME49_246178-Fw	GCCTGAACTTTGTGGACTCAC	Detect the replacement of C-terminal TGME49_246178 by 6×HA fragment in PCR2
PCR2-TGME49_246178-Rv	GGCGGGAGAAGGTAGAGATC	Detect the replacement of C-terminal TGME49_246178 by 6×HA fragment in PCR2
sgRNA-TGME49_254070-Tag	TGCACACATCAGCTCCTTTC	Designed SgRNA-Tag sequence for TGME49_254070
sgRNA-TGME49_254070-Tag-Fw	TGCACACATCAGCTCCTTTCGTTTTAGAGCTAGAAATAGC	Construct the CRISPR plasmid for tagging TGME49_254070 with 6×HA
TGME49_254070-HR-Fw	CCGGGCTTTGTGCAGCAATGCGTCACAAATCCCGAAAGGAGCGCTAGCAAGGGCTCGGG	Amplify 6HA-DHFR fragment with the homologous fragment of *TGME49_254070*
TGME49_254070-HR-Rv	CATTTGCGATACTTCTTGCACACATCAGCTCCTTTCGGGATTATACGACTCACTATAGG	Amplify 6HA-DHFR fragment with the homologous fragment of *TGME49_254070*

PCR1/2-TGME49_254070-Fw	CACTCACGAGGCAGTGAG	Detect the replacement of C-terminal TGME49_254070 by 6×HA fragment in PCR2
PCR2-TGME49_254070-Rv	CAAATGTGATCACGGTACGC	Detect the replacement of C-terminal TGME49_254070 by 6×HA fragment in PCR2
sgRNA-TGME49_254880-Tag	CGCATGCCAGCTGAGGAAAA	Designed SgRNA-Tag sequence for TGME49_254880
sgRNA-TGME49_254880-Tag-Fw	CGCATGCCAGCTGAGGAAAAGTTTTAGAGCTAGAAATAGC	Construct the CRISPR plasmid for tagging TGME49_254880 with 6×HA
TGME49_254880-HR-Fw	GCCCGGATGCAGCTGATGCAGTGCGAGAGGCCGCATGCCAGCGCTAGCAAGGGCTCGGG	Amplify 6HA-DHFR fragment with the homologous fragment of *TGME49_254880*
TGME49_254880-HR-Rv	TTCGACTCGCCAGAAAAAACTCCCACGGGTCACTTGCCATTTATACGACTCACTATAGG	Amplify 6HA-DHFR fragment with the homologous fragment of *TGME49_254880*
PCR1/2-TGME49_254880-Fw	CTCGTCAAGCGTGGAGAAG	Detect the replacement of C-terminal TGME49_254880 by 6×HA fragment in PCR2
PCR2-TGME49_254880-Rv	GTACCGTTGTCGTTGAACTC	Detect the replacement of C-terminal TGME49_254880 by 6×HA fragment in PCR2
sgRNA-TGME49_264600-Tag	TCTGCCCAGGCGTAGAAGAC	Designed SgRNA-Tag sequence for TGME49_264600
sgRNA-TGME49_264600-Tag-Fw	TCTGCCCAGGCGTAGAAGACGTTTTAGAGCTAGAAATAGC	Construct the CRISPR plasmid for tagging TGME49_264600 with 6×HA
TGME49_264600-HR-Fw	CTACACAAAGCACTGCCTGCTGGAGATGCGTCTGCCCAGGCGGCTAGCAAGGGCTCGGG	Amplify 6HA-DHFR fragment with the homologous fragment of *TGME49_264600*
TGME49_264600-HR-Rv	GAAAGCAGCCGCTGCTGCCGCAACAAAATCATCGTGCCTGTCATACGACTCACTATAGG	Amplify 6HA-DHFR fragment with the homologous fragment of *TGME49_264600*
PCR1/2-TGME49_264600-Fw	GCCAGTCGATCGGAAGTAC	Detect the replacement of C-terminal TGME49_264600 by 6×HA fragment in PCR2
PCR2-TGME49_264600-Rv	GCTTTGGCACAGAGACAACG	Detect the replacement of C-terminal TGME49_264600 by 6×HA fragment in PCR2
sgRNA-TGME49_270200-Tag	AAGCAGTGAAGTGCGTGTTT	Designed SgRNA-Tag sequence for TGME49_270200
sgRNA-TGME49_270200-Tag-Fw	AAGCAGTGAAGTGCGTGTTTGTTTTAGAGCTAGAAATAGC	Construct the CRISPR plasmid for tagging TGME49_270200 with 6×HA
TGME49_270200-HR-Fw	GGAGCAGATGCAGATCTTATTTGGGGAAAGCGTAGAAAGCAGGCTAGCAAGGGCTCGGG	Amplify 6HA-DHFR fragment with the homologous fragment of *TGME49_270200*
TGME49_270200-HR-Rv	ATTGTCAAACGTCTACACGTTTACTCCACCTTTTTTCCAAAAATACGACTCACTATAGG	Amplify 6HA-DHFR fragment with the homologous fragment of *TGME49_270200*
PCR1/2-TGME49_270200-Fw	GCTGCTGAGTATCCAAGTGG	Detect the replacement of C-terminal TGME49_270200 by 6×HA fragment in PCR2
PCR2-TGME49_270200-Rv	GAACCAGGAACACAGCATGC	Detect the replacement of C-terminal TGME49_270200 by 6×HA fragment in PCR2

sgRNA-TGME49_271270-Tag	GCAGGTGACACGGCTGTCTA	Designed SgRNA-Tag sequence for TGME49_271270
sgRNA-TGME49_271270-Tag-Fw	GCAGGTGACACGGCTGTCTAGTTTTAGAGCTAGAAATAGC	Construct the CRISPR plasmid for tagging TGME49_271270 with 6×HA
TGME49_271270-HR-Fw	TGTCTGTGCATGTGCCGGATGATGTATCTTCTCAAAAGCAGGGCTAGCAAGGGCTCGGG	Amplify 6HA-DHFR fragment with the homologous fragment of *TGME49_271270*
TGME49_271270-HR-Rv	GCGCCATCGGGAGCCTCAGTTCTTTAGACATAACTGCCTTAGATACGACTCACTATAGG	Amplify 6HA-DHFR fragment with the homologous fragment of *TGME49_271270*
PCR1/2-TGME49_271270-Fw	GTATCTGTGGCACCGAGTTG	Detect the replacement of C-terminal TGME49_271270 by 6×HA fragment in PCR2
PCR2-TGME49_271270-Rv	GAAGTCAAGTGCGACGTCTC	Detect the replacement of C-terminal TGME49_271270 by 6×HA fragment in PCR2
sgRNA-TGME49_273860-Tag	CGTCAAAAGATCACAATTAT	Designed SgRNA-Tag sequence for TGME49_273860
sgRNA-TGME49_273860-Tag-Fw	CGTCAAAAGATCACAATTATGTTTTAGAGCTAGAAATAGC	Construct the CRISPR plasmid for tagging TGME49_273860 with 6×HA
TGME49_273860-HR-Fw	GAGGACAAATTTTTACATCAAATACACGTGGAGAATCGAAGCGCTAGCAAGGGCTCGGG	Amplify 6HA-DHFR fragment with the homologous fragment of *TGME49_273860*
TGME49_273860-HR-Rv	CTCCGTAAGGAACACAGGCTTCATTGGACGGTCTGGCCCATAATACGACTCACTATAGG	Amplify 6HA-DHFR fragment with the homologous fragment of *TGME49_273860*
PCR1/2-TGME49_273860-Fw	GCGATAAGCACGATACAGAGG	Detect the replacement of C-terminal TGME49_273860 by 6×HA fragment in PCR2
PCR2-TGME49_273860-Rv	GCGTGACATTACGCACATG	Detect the replacement of C-terminal TGME49_273860 by 6×HA fragment in PCR2
sgRNA-TGME49_279420-Tag	ATTAGAAAGCCGTCGTCGACG	Designed SgRNA-Tag sequence for TGME49_279420
sgRNA-TGME49_279420-Tag-Fw	ATTAGAAAGCCGTCGTCGACGTTTTAGAGCTAGAAATAGC	Construct the CRISPR plasmid for tagging TGME49_279420 with 6×HA
TGME49_279420-HR-Fw	TCAGATATTGAAGAGGAATCGGAAAACGGGAAAATCGGCTATGCTAGCAAGGGCTCGGG	Amplify 6HA-DHFR fragment with the homologous fragment of *TGME49_279420*
TGME49_279420-HR-Rv	CTGCAGCCTTGCTCCTCGCCTCATGAACCTCCCGCACCAGTCATACGACTCACTATAGG	Amplify 6HA-DHFR fragment with the homologous fragment of *TGME49_279420*
PCR1/2-TGME49_279420-Fw	GAAGACGTCATGAAGCAGATG	Detect the replacement of C-terminal TGME49_279420 by 6×HA fragment in PCR2
PCR2-TGME49_279420-Rv	GAGACACTGCAGTAGTACTCG	Detect the replacement of C-terminal TGME49_279420 by 6×HA fragment in PCR2
sgRNA-TGME49_305270-Tag	TACGGTCACTCCTGCGCAAA	Designed SgRNA-Tag sequence for TGME49_305270
sgRNA-TGME49_305270-Tag-Fw	TACGGTCACTCCTGCGCAAAGTTTTAGAGCTAGAAATAGC	Construct the CRISPR plasmid for tagging TGME49_305270 with 6×HA

TGME49_305270-HR-Fw	GGCACATCGTTGAGAATCCTCCAGTCGCCGTTTGCGCAGGAGGCTAGCAAGGGCTCGGG	Amplify 6HA-DHFR fragment with the homologous fragment of *TGME49_305270*
TGME49_305270-HR-Rv	CAAGGAAAAAAGTTATTACGGTCACTCCTGCGCAAACGGCGAATACGACTCACTATAGG	Amplify 6HA-DHFR fragment with the homologous fragment of *TGME49_305270*
PCR1/2-TGME49_305270-Fw	GATCTCGACTGCTGTGACAC	Detect the replacement of C-terminal TGME49_305270 by 6×HA fragment in PCR2
PCR2-TGME49_305270-Rv	CCGTCGTACACCTCGTTGA	Detect the replacement of C-terminal TGME49_305270 by 6×HA fragment in PCR2
sgRNA-TGME49_306895-Tag	TGGCTAGTGGAGGCTCGATC	Designed SgRNA-Tag sequence for TGME49_306895
sgRNA-TGME49_306895-Tag-Fw	TGGCTAGTGGAGGCTCGATCGTTTTAGAGCTAGAAATAGC	Construct the CRISPR plasmid for tagging TGME49_306895 with 6×HA
TGME49_306895-HR-Fw	ACGCCTTTAACAATTGACCAGGTCACCGGTGGAGATGTCGCCGCTAGCAAGGGCTCGGG	Amplify 6HA-DHFR fragment with the homologous fragment of *TGME49_306895*
TGME49_306895-HR-Rv	TTGCACCGGTTTTGTCTGGCTAGTGGAGGCTCGATCAGGCGAATACGACTCACTATAGG	Amplify 6HA-DHFR fragment with the homologous fragment of *TGME49_306895*
PCR1/2-TGME49_306895-Fw	CCAGCAATGAGGTGGTTGAC	Detect the replacement of C-terminal TGME49_306895 by 6×HA fragment in PCR2
PCR2-TGME49_306895-Rv	GTTCCTTGGAGCATGACACAC	Detect the replacement of C-terminal TGME49_306895 by 6×HA fragment in PCR2
