# Supplementary material for: Functional characterization of 11 novel rhoptry proteins in the type I RH strain of Toxoplasma gondii using the CRISPR-Cas9 system
Source: Parasit Vectors. 2026 Apr 13;19:221. doi: 10.1186/s13071-026-07387-0 (PMC13185275; doi:10.1186/s13071-026-07387-0)
Supplement: Supplementary file 2 — Additional file 2. Table S2. Primers employed for generating gene knockout strains. [file 13071_2026_7387_MOESM2_ESM.pdf]

Additional file 2: Table S2 Primers employed for generating gene knockout strains.

Primer	Sequence (5′→3′)	Use
DHFR-Gibson-Fw	AAGCTTCGCCAGGCTGTAAATCC	Amplify the DHFR fragment to construct the pUPRT-DHFR-D plasmid
DHFR-Gibson-Rv	GAATTCATCCTGCAAGTGCATAG	Amplify the DHFR fragment to construct the pUPRT-DHFR-D plasmid
pUC19-Gibson-Fw	TGTGAAATTGTTATCCGCTC	Amplify the pUC19 fragment to construct the pUPRT-DHFR-D plasmid
pUC19-Gibson-Rv	AACGTCGTGACTGGGAAAACC	Amplify the pUC19 fragment to construct the pUPRT-DHFR-D plasmid
sgRNA-KO-Rv	AACTTGACATCCCCATTTAC	Construct the CRISPR plasmid for deleting ROP genes (universal primer)
PCR3-DHFR-Rv	GCCAAAGTAGAAAGGAATTAGCAT	Detect the insertion of 5' homologous fragment of ROP genes in PCR3 (universal primer)
PCR5-DHFR-Fw	TGACGCAGATGTGCGTGTATCCAC	Detect the insertion of 3' homologous fragment of ROP genes in PCR5 (universal primer)
sgRNA-TGME49_312150-KO	GCAGAAGGAATTCTCGGCCA	Designed SgRNA-KO sequence for TGME49_312150
sgRNA-TGME49_312150-KO-Fw	GCAGAAGGAATTCTCGGCCAGTTTTAGAGCTAGAAATAGC	Construct the CRISPR plasmid for deleting TGME49_312150
U5-TGME49_312150-Fw	GGTTTTCCCAGTCACGACGTTGTGAAGCAAGTGCGATAGATG	Amplify the 5' homologous arms of TGME49_312150 to construct the pUPRT-DHFR-D plasmid
U5-TGME49_312150-Rv	GGATTTACAGCCTGGCGAAGCTTCCATTTTTCGTGCGTGCAAG	Amplify the 5' homologous arms of TGME49_312150 to construct the pUPRT-DHFR-D plasmid
U3-TGME49_312150-Fw	CTATGCACTTGCAGGATGAATTCGTCGTTGTCACATCAGACTG	Amplify the 3' homologous arms of TGME49_312150 to construct the pUPRT-DHFR-D plasmid
U3-TGME49_312150-Rv	GAGCGGATAACAATTTCACACAACATGCAGGTCAGTTGTC	Amplify the 3' homologous arms of TGME49_312150 to construct the pUPRT-DHFR-D plasmid
PCR3-TGME49_312150-Fw	GTGTGACAAGTGCAACAGAG	Detect the insertion of 5' homologous fragment of TGME49_312150 in PCR3
PCR4-TGME49_312150-Fw	GTGACCATGCCATTCTCTTG	Detect the deletion of TGME49_312150 in PCR4
PCR4-TGME49_312150-Rv	CAGACGCATTCAACTTACTGT	Detect the deletion of TGME49_312150 in PCR4
PCR5-TGME49_312150-Rv	GCTGTGTGTCGAAACATCTC	Detect the insertion of 3' homologous fragment of TGME49_312150 in PCR5
sgRNA-TGME49_246178-KO	GCTACTGGATCCGCTTCCGG	Designed SgRNA-KO sequence for TGME49_246178

sgRNA-TGME49_246178-KO-Fw	GCTACTGGATCCGCTTCCGGGTTTTAGAGCTAGAAATAGC	Construct the CRISPR plasmid for deleting TGME49_246178
U5-TGME49_246178-Fw	GGTTTTCCCAGTCACGACGTTGAACCGTCCTTGTGATCGTC	Amplify the 5' homologous arms of TGME49_246178 to construct the pUPRT-DHFR-D plasmid
U5-TGME49_246178-Rv	GGATTTACAGCCTGGCGAAGCTTGGACGAGACAGCTGATGATC	Amplify the 5' homologous arms of TGME49_246178 to construct the pUPRT-DHFR-D plasmid
U3-TGME49_246178-Fw	CTATGCACTTGCAGGATGAATTCGACGAAGACGTCGAACACG	Amplify the 3' homologous arms of TGME49_246178 to construct the pUPRT-DHFR-D plasmid
U3-TGME49_246178-Rv	GAGCGGATAACAATTTCACAGCCTCATGTGTGGTTTGCTG	Amplify the 3' homologous arms of TGME49_246178 to construct the pUPRT-DHFR-D plasmid
PCR3-TGME49_246178-Fw	GCATCTCCTTTCGCAGCTTC	Detect the insertion of 5' homologous fragment of TGME49_246178 in PCR3
PCR4-TGME49_246178-Fw	GACTTGTGTATCTCGAGTGCG	Detect the deletion of TGME49_246178 in PCR4
PCR4-TGME49_246178-Rv	GCTTCGATTTGAGGAGCCAC	Detect the deletion of TGME49_246178 in PCR4
PCR5-TGME49_246178-Rv	GTACGAGGGAAGTCTGCAAG	Detect the insertion of 3' homologous fragment of TGME49_246178 in PCR5
sgRNA-TGME49_254070-KO	GCGCTCGGACAACGTCAACG	Designed SgRNA-KO sequence for TGME49_254070
sgRNA-TGME49_254070-KO-Fw	GCGCTCGGACAACGTCAACGGTTTTAGAGCTAGAAATAGC	Construct the CRISPR plasmid for deleting TGME49_254070
U5-TGME49_254070-Fw	GGTTTTCCCAGTCACGACGTTGACTAGCGTAGCGGTTACTTC	Amplify the 5' homologous arms of TGME49_254070 to construct the pUPRT-DHFR-D plasmid
U5-TGME49_254070-Rv	GGATTTACAGCCTGGCGAAGCTTGATGTAAACCACCGCTGCTC	Amplify the 5' homologous arms of TGME49_254070 to construct the pUPRT-DHFR-D plasmid
U3-TGME49_254070-Fw	CTATGCACTTGCAGGATGAATTCAGCGTGTAGCGTCGTAAAGC	Amplify the 3' homologous arms of TGME49_254070 to construct the pUPRT-DHFR-D plasmid
U3-TGME49_254070-Rv	GAGCGGATAACAATTTCACAGTGTCACCAGGGCGATATG	Amplify the 3' homologous arms of TGME49_254070 to construct the pUPRT-DHFR-D plasmid
PCR3-TGME49_254070-Fw	GTCAGTCCTGGACTCGAGAG	Detect the insertion of 5' homologous fragment of TGME49_254070 in PCR3
PCR4-TGME49_254070-Fw	GGCAGTAGACTAGATTGCGC	Detect the deletion of TGME49_254070 in PCR4
PCR4-TGME49_254070-Rv	CCATTGGTGAATTCAGTTGCC	Detect the deletion of TGME49_254070 in PCR4
PCR5-TGME49_254070-Rv	GAGAGGCAGCTACAAGTCAG	Detect the insertion of 3' homologous fragment of TGME49_254070 in PCR5
sgRNA-TGME49_254880-KO	GGACAGCATGAAGAAGATGG	Designed SgRNA-KO sequence for TGME49_254880

sgRNA-TGME49_254880-KO-Fw	GGACAGCATGAAGAAGATGGGTTTTAGAGCTAGAAATAGC	Construct the CRISPR plasmid for deleting TGME49_254880
U5-TGME49_254880-Fw	GGTTTTCCCAGTCACGACGTTGGTACAGCTAGCTGGTAGGAG	Amplify the 5' homologous arms of TGME49_254880 to construct the pUPRT-DHFR-D plasmid
U5-TGME49_254880-Rv	GGATTTACAGCCTGGCGAAGCTTCACGAACCGGCTCGAATG	Amplify the 5' homologous arms of TGME49_254880 to construct the pUPRT-DHFR-D plasmid
U3-TGME49_254880-Fw	CTATGCACTTGCAGGATGAATTCGTGAACAGCCAGAGGCAAC	Amplify the 3' homologous arms of TGME49_254880 to construct the pUPRT-DHFR-D plasmid
U3-TGME49_254880-Rv	GAGCGGATAACAATTTCACAGAAGAGAAGCGTCTCTCCTC	Amplify the 3' homologous arms of TGME49_254880 to construct the pUPRT-DHFR-D plasmid
PCR3-TGME49_254880-Fw	GACGCTGCAGACTCAAGGTC	Detect the insertion of 5' homologous fragment of TGME49_254880 in PCR3
PCR4-TGME49_254880-Fw	GTTGCTGGCCTAGAACGTATC	Detect the deletion of TGME49_254880 in PCR4
PCR4-TGME49_254880-Rv	GACTAGCTGCTATGTAGGACG	Detect the deletion of TGME49_254880 in PCR4
PCR5-TGME49_254880-Rv	GTGGCTCGTCTACCGTTCAG	Detect the insertion of 3' homologous fragment of TGME49_254880 in PCR5
sgRNA-TGME49_264600-KO	GTTAATGCTTGTGTGAACCG	Designed SgRNA-KO sequence for TGME49_264600
sgRNA-TGME49_264600-KO-Fw	GTTAATGCTTGTGTGAACCGGTTTTAGAGCTAGAAATAGC	Construct the CRISPR plasmid for deleting TGME49_264600
U5-TGME49_264600-Fw	GGTTTTCCCAGTCACGACGTTGTAATTCTCGGAGCCTCTGC	Amplify the 5' homologous arms of TGME49_264600 to construct the pUPRT-DHFR-D plasmid
U5-TGME49_264600-Rv	GGATTTACAGCCTGGCGAAGCTTGTTTTCCTGCAGGATGTCCG	Amplify the 5' homologous arms of TGME49_264600 to construct the pUPRT-DHFR-D plasmid
U3-TGME49_264600-Fw	CTATGCACTTGCAGGATGAATTCGCTCATGAACGTGCTACAC	Amplify the 3' homologous arms of TGME49_264600 to construct the pUPRT-DHFR-D plasmid
U3-TGME49_264600-Rv	GAGCGGATAACAATTTCACAGACTGTCGTTGATAGTGGCTG	Amplify the 3' homologous arms of TGME49_264600 to construct the pUPRT-DHFR-D plasmid
PCR3-TGME49_264600-Fw	GTACCTGGAATGTCTCGTGC	Detect the insertion of 5' homologous fragment of TGME49_264600 in PCR3
PCR4-TGME49_264600-Fw	GTACATTCGCCTGGAGGTG	Detect the deletion of TGME49_264600 in PCR4
PCR4-TGME49_264600-Rv	GCGATGTTGCAGAGTCGTC	Detect the deletion of TGME49_264600 in PCR4
PCR5-TGME49_264600-Rv	GCTAGGTGTCACGGAACAAG	Detect the insertion of 3' homologous fragment of TGME49_264600 in PCR5
sgRNA-TGME49_270200-KO	GGAATCTGCGATTGAAGCGT	Designed SgRNA-KO sequence for TGME49_270200

sgRNA-TGME49_270200-KO-Fw	GGAATCTGCGATTGAAGCGTGTTTTAGAGCTAGAAATAGC	Construct the CRISPR plasmid for deleting TGME49_270200
U5-TGME49_270200-Fw	GGTTTTCCCAGTCACGACGTTGCAAGTGTGAGAAGGCAAGC	Amplify the 5' homologous arms of TGME49_270200 to construct the pUPRT-DHFR-D plasmid
U5-TGME49_270200-Rv	GGATTTACAGCCTGGCGAAGCTTGACTCGCGCGAATATTGTCC	Amplify the 5' homologous arms of TGME49_270200 to construct the pUPRT-DHFR-D plasmid
U3-TGME49_270200-Fw	CTATGCACTTGCAGGATGAATTCGGTTGGAGCAGATGCAGATC	Amplify the 3' homologous arms of TGME49_270200 to construct the pUPRT-DHFR-D plasmid
U3-TGME49_270200-Rv	GAGCGGATAACAATTTCACAGGCAAGGTATCGCATATACG	Amplify the 3' homologous arms of TGME49_270200 to construct the pUPRT-DHFR-D plasmid
PCR3-TGME49_270200-Fw	GCTGTCGTCATCGCATATACC	Detect the insertion of 5' homologous fragment of TGME49_270200 in PCR3
PCR4-TGME49_270200-Fw	GACTGGTCGTCGGTCTGTTC	Detect the deletion of TGME49_270200 in PCR4
PCR4-TGME49_270200-Rv	GAGATGAAGCGAGCACAGC	Detect the deletion of TGME49_270200 in PCR4
PCR5-TGME49_270200-Rv	GTGTCAGCGTTCTGACTGTG	Detect the insertion of 3' homologous fragment of TGME49_270200 in PCR5
sgRNA-TGME49_271270-KO	GCATGTCGACTCAACACAGG	Designed SgRNA-KO sequence for TGME49_271270
sgRNA-TGME49_271270-KO-Fw	GCATGTCGACTCAACACAGGGTTTTAGAGCTAGAAATAGC	Construct the CRISPR plasmid for deleting TGME49_271270
U5-TGME49_271270-Fw	GGTTTTCCCAGTCACGACGTTGAAGATATGCCAGCGACCAC	Amplify the 5' homologous arms of TGME49_271270 to construct the pUPRT-DHFR-D plasmid
U5-TGME49_271270-Rv	GGATTTACAGCCTGGCGAAGCTTGCACTGATGCCACAGGTAG	Amplify the 5' homologous arms of TGME49_271270 to construct the pUPRT-DHFR-D plasmid
U3-TGME49_271270-Fw	CTATGCACTTGCAGGATGAATTCGCTCGCTTGTCTGTGCATG	Amplify the 3' homologous arms of TGME49_271270 to construct the pUPRT-DHFR-D plasmid
U3-TGME49_271270-Rv	GAGCGGATAACAATTTCACAGCATCAGTGCATCTAGCTTG	Amplify the 3' homologous arms of TGME49_271270 to construct the pUPRT-DHFR-D plasmid
PCR3-TGME49_271270-Fw	GCTCTTGGTCACTTGAAGGT	Detect the insertion of 5' homologous fragment of TGME49_271270 in PCR3
PCR4-TGME49_271270-Fw	GTCTACCTGCAGCGTATATGG	Detect the deletion of TGME49_271270 in PCR4
PCR4-TGME49_271270-Rv	GTGCGTGAAGGTAATGTGTC	Detect the deletion of TGME49_271270 in PCR4
PCR5-TGME49_271270-Rv	GAGTCTGGTGAGTCTCTGAG	Detect the insertion of 3' homologous fragment of TGME49_271270 in PCR5
sgRNA-TGME49_273860-KO	GATGAAGATTCGGCAGAAGG	Designed SgRNA-KO sequence for TGME49_273860

sgRNA-TGME49_273860-KO-Fw	GATGAAGATTCGGCAGAAGGGTTTTAGAGCTAGAAATAGC	Construct the CRISPR plasmid for deleting TGME49_273860
U5-TGME49_273860-Fw	GGTTTTCCCAGTCACGACGTTGTGTGAGCAGAGTCACCATTG	Amplify the 5' homologous arms of TGME49_273860 to construct the pUPRT-DHFR-D plasmid
U5-TGME49_273860-Rv	GGATTTACAGCCTGGCGAAGCTTGGACGAAGAAACCTGGTTACG	Amplify the 5' homologous arms of TGME49_273860 to construct the pUPRT-DHFR-D plasmid
U3-TGME49_273860-Fw	CTATGCACTTGCAGGATGAATTCTACACGTGGAGAATCGAAGC	Amplify the 3' homologous arms of TGME49_273860 to construct the pUPRT-DHFR-D plasmid
U3-TGME49_273860-Rv	GAGCGGATAACAATTTCACAGGTGTAGGCAGATGGATACAC	Amplify the 3' homologous arms of TGME49_273860 to construct the pUPRT-DHFR-D plasmid
PCR3-TGME49_273860-Fw	GGACGTCAGGATCTATACCAG	Detect the insertion of 5' homologous fragment of TGME49_273860 in PCR3
PCR4-TGME49_273860-Fw	GCCAGCAGAATGTACTATGTC	Detect the deletion of TGME49_273860 in PCR4
PCR4-TGME49_273860-Rv	GCACCTTGTATTTACCGCTAG	Detect the deletion of TGME49_273860 in PCR4
PCR5-TGME49_273860-Rv	GCATATCATACGCGTTCCG	Detect the insertion of 3' homologous fragment of TGME49_273860 in PCR5
sgRNA-TGME49_279420-KO	GCAGCTGAGGAGGCACGCGA	Designed SgRNA-KO sequence for TGME49_279420
sgRNA-TGME49_279420-KO-Fw	GCAGCTGAGGAGGCACGCGAGTTTTAGAGCTAGAAATAGC	Construct the CRISPR plasmid for deleting TGME49_279420
U5-TGME49_279420-Fw	GGTTTTCCCAGTCACGACGTTGTGAGTGCTCTCCAACATCC	Amplify the 5' homologous arms of TGME49_279420 to construct the pUPRT-DHFR-D plasmid
U5-TGME49_279420-Rv	GGATTTACAGCCTGGCGAAGCTTGAGCTGAAGCGAAGACATGC	Amplify the 5' homologous arms of TGME49_279420 to construct the pUPRT-DHFR-D plasmid
U3-TGME49_279420-Fw	CTATGCACTTGCAGGATGAATTCGGTAGCAGCACAACCAATAG	Amplify the 3' homologous arms of TGME49_279420 to construct the pUPRT-DHFR-D plasmid
U3-TGME49_279420-Rv	GAGCGGATAACAATTTCACAGATACAGTAGCTCAGCACATG	Amplify the 3' homologous arms of TGME49_279420 to construct the pUPRT-DHFR-D plasmid
PCR3-TGME49_279420-Fw	GTGGATGTCGGTCATGGAC	Detect the insertion of 5' homologous fragment of TGME49_279420 in PCR3
PCR4-TGME49_279420-Fw	CATGAGCATGTCTTCGCTTC	Detect the deletion of TGME49_279420 in PCR4
PCR4-TGME49_279420-Rv	GGATTCAATCTGGTTGCTGC	Detect the deletion of TGME49_279420 in PCR4
PCR5-TGME49_279420-Rv	GCGACAATGAACACACCAC	Detect the insertion of 3' homologous fragment of TGME49_279420 in PCR5
sgRNA-TGME49_305270-KO	GTTCCGCCTGAATCCACCGA	Designed SgRNA-KO sequence for TGME49_305270

sgRNA-TGME49_305270-KO-Fw	GTTCCGCCTGAATCCACCGAGTTTTAGAGCTAGAAATAGC	Construct the CRISPR plasmid for deleting TGME49_305270
U5-TGME49_305270-Fw	GGTTTTCCCAGTCACGACGTTGTTCCTTCGTGTGTCTTTGC	Amplify the 5' homologous arms of TGME49_305270 to construct the pUPRT-DHFR-D plasmid
U5-TGME49_305270-Rv	GGATTTACAGCCTGGCGAAGCTTGGAACAAGGCAAGCAAGAG	Amplify the 5' homologous arms of TGME49_305270 to construct the pUPRT-DHFR-D plasmid
U3-TGME49_305270-Fw	CTATGCACTTGCAGGATGAATTCCATCGTTGAGAATCCTCCAG	Amplify the 3' homologous arms of TGME49_305270 to construct the pUPRT-DHFR-D plasmid
U3-TGME49_305270-Rv	GAGCGGATAACAATTTCACACAGTCGATTCCGATAACGATC	Amplify the 3' homologous arms of TGME49_305270 to construct the pUPRT-DHFR-D plasmid
PCR3-TGME49_305270-Fw	CGCACATCAGTGACTTATCTC	Detect the insertion of 5' homologous fragment of TGME49_305270 in PCR3
PCR4-TGME49_305270-Fw	GGTCGGGCGTAAGTTTCA	Detect the deletion of TGME49_305270 in PCR4
PCR4-TGME49_305270-Rv	TGTTGTCCAGCGGTGTTT	Detect the deletion of TGME49_305270 in PCR4
PCR5-TGME49_305270-Rv	GGACAACACTCAAGTCACGAG	Detect the insertion of 3' homologous fragment of TGME49_305270 in PCR5
sgRNA-TGME49_306895-KO	GTCAACCACCTCATTGCTGG	Designed SgRNA-KO sequence for TGME49_306895
sgRNA-TGME49_306895-KO-Fw	GTCAACCACCTCATTGCTGGGTTTTAGAGCTAGAAATAGC	Construct the CRISPR plasmid for deleting TGME49_306895
U5-TGME49_306895-Fw	GGTTTTCCCAGTCACGACGTTGACGTAACTGGTGGACCTCG	Amplify the 5' homologous arms of TGME49_306895 to construct the pUPRT-DHFR-D plasmid
U5-TGME49_306895-Rv	GGATTTACAGCCTGGCGAAGCTTGTGTTGATTGAGCAGTGTCC	Amplify the 5' homologous arms of TGME49_306895 to construct the pUPRT-DHFR-D plasmid
U3-TGME49_306895-Fw	CTATGCACTTGCAGGATGAATTCGCACTGCACATCTTCCTCTC	Amplify the 3' homologous arms of TGME49_306895 to construct the pUPRT-DHFR-D plasmid
U3-TGME49_306895-Rv	GAGCGGATAACAATTTCACAGGCACTCTAGTGTACTCTACG	Amplify the 3' homologous arms of TGME49_306895 to construct the pUPRT-DHFR-D plasmid
PCR3-TGME49_306895-Fw	CAGACAGTAGTCAGCAAGCG	Detect the insertion of 5' homologous fragment of TGME49_306895 in PCR3
PCR4-TGME49_306895-Fw	GAGTTAGCTGTGCACTGTCA	Detect the deletion of TGME49_306895 in PCR4
PCR4-TGME49_306895-Rv	GCTAGTGGAGGCTCGATCAG	Detect the deletion of TGME49_306895 in PCR4
PCR5-TGME49_306895-Rv	GAGCACATCGTCACATGC	Detect the insertion of 3' homologous fragment of TGME49_306895 in PCR5
