# Supplementary material for: Functional characterization of 11 novel rhoptry proteins in the type I RH strain of Toxoplasma gondii using the CRISPR-Cas9 system
Source: Parasit Vectors. 2026 Apr 13;19:221. doi: 10.1186/s13071-026-07387-0 (PMC13185275; doi:10.1186/s13071-026-07387-0)
Supplement: Supplementary file 3 — Additional file 3. Table S3. Differentially expressed ROP, GRA, and MIC genes uniquely identified in the RHΔrop64 strain. [file 13071_2026_7387_MOESM3_ESM.pdf]

Additional file 3: Table S3 Differentially expressed ROP, GRA, and MIC genes uniquely identified in the RHΔ*rop64* strain.

**Gene ID**	**Gene type**	**Product description**	**Regulation**
TGME49_205360	ROP	Hypothetical protein	Down
TGME49_209985	ROP	Rhoptry protein ROP42	Down
TGME49_211290	ROP	Rhoptry protein ROP15	Down
TGME49_222100	ROP	Effector protein SOS1	Down
TGME49_223920	ROP	Rhoptry neck protein RON3	Down
TGME49_237180	ROP	Hypothetical protein	Down
TGME49_273860	ROP	Hypothetical protein	Down
TGME49_279420	ROP	Hypothetical protein	Down
TGME49_282055	ROP	Protein phosphatase PP2C-hn	Down
TGME49_291960	ROP	Rhoptry kinase family protein ROP40 (incomplete catalytic triad)	Down
TGME49_297960	ROP	Rhoptry neck protein RON6	Down
TGME49_306060	ROP	Rhoptry neck protein RON8	Down
TGME49_311470	ROP	Rhoptry neck protein RON5	Down
TGME49_312270	ROP	Rhoptry protein ROP13	Down
TGME49_218270	ROP	Rhoptry protein ROP48	Down
TGME49_225320	ROP	Hypothetical protein	Down
TGME49_230350	ROP	Rhoptry neck protein RON11	Down
TGME49_232020	ROP	Rhoptry neck protein RON12	Down
TGME49_242118	ROP	Myosin-light-chain kinase	Down
TGME49_271270	ROP	Hypothetical protein	Down
TGME49_310740	ROP	Hypothetical protein	Down
TGME49_315210	ROP	Rhoptry protein, putative	Down
TGME49_281675	ROP	Rhoptry kinase family protein ROP35	Down
TGME49_203310	GRA	Dense granule protein GRA7	Up
TGME49_208450	GRA	Protease inhibitor PI2	Up
TGME49_213050	GRA	Hypothetical protein	Up
TGME49_215910	GRA	Hypothetical protein	Up
TGME49_220950	GRA	Hypothetical protein	Up
TGME49_251540	GRA	Dense granule protein GRA9	Up
TGME49_254720	GRA	Dense granule protein GRA8	Up

TGME49_279100	GRA	Mitochondrial association factor 1a	Up
TGME49_289050	GRA	FIKK kinase, putative	Up
TGME49_294940	GRA	Hypothetical protein	Up
TGME49_294970	GRA	Hypothetical protein	Up
TGME49_320490	GRA	Dense granule protein GRA66	Up
TGME49_208830	GRA	Dense granule protein GRA16	Down
TGME49_212410	GRA	Dense granule protein GRA11A	Down
TGME49_239010	GRA	E2F4-associated EZH2-inducing gene regulator TEEGR	Down
TGME49_239752	GRA	Hypothetical protein	Down
TGME49_269460	GRA	Basal complex component BCC5	Down
TGME49_277270	GRA	NTPase II	Down
TGME49_301480	GRA	Hypothetical protein	Down
TGME49_200240	MIC	Microneme protein MIC17B	Up
TGME49_277080	MIC	Microneme protein MIC5	Up
TGME49_292020	MIC	Cysteine repeat modular protein CRMPB	Down
