# Supplementary material for: Functional characterization of 11 novel rhoptry proteins in the type I RH strain of Toxoplasma gondii using the CRISPR-Cas9 system
Source: Parasit Vectors. 2026 Apr 13;19:221. doi: 10.1186/s13071-026-07387-0 (PMC13185275; doi:10.1186/s13071-026-07387-0)
Supplement: Supplementary file 4 — Additional file 4. Table S4. Differentially expressed ROP, GRA, and MIC genes uniquely identified in the RHΔrop65 strain. [file 13071_2026_7387_MOESM4_ESM.pdf]

Additional file 4: Table S4 Differentially expressed ROP, GRA, and MIC genes uniquely identified in the RHΔ*rop65* strain.

**Gene ID**	**Gene type**	**Product description**	**Regulation**
TGME49_254070	ROP	Hypothetical protein	Up
TGME49_225200	ROP	Hypothetical protein	Down
TGME49_277720	ROP	GDA1/CD39 (nucleoside phosphatase) family protein	Down
TGME49_305270	ROP	Hypothetical protein	Down
TGME49_203600	GRA	Dense granule protein GRA50	Up
TGME49_211460	GRA	Myc-regulation protein MYR4	Up
TGME49_224460	GRA	Aminopeptidase N3	Up
TGME49_227280	GRA	Dense granule protein GRA3	Up
TGME49_237500	GRA	Protein phosphatase PPM3A, putative	Up
TGME49_297880	GRA	Dense granule protein GRA23	Up
TGME49_306890	GRA	Hypothetical protein	Up
TGME49_317705	GRA	Enoyl-CoA hydratase/isomerase family protein	Up
TGME49_217680	GRA	Dense granule protein GRA57	Down
TGME49_247220	GRA	Nudix -type motif 9 isoform a family protein	Down
TGME49_200250	MIC	Microneme protein MIC17A	Up
TGME49_201780	MIC	Microneme protein MIC2	Down
TGME49_206510	MIC	Toxolysin TLN4	Down
TGME49_283540	MIC	Microneme protein MIC20	Down
