# Supplementary material for: Functional characterization of 11 novel rhoptry proteins in the type I RH strain of Toxoplasma gondii using the CRISPR-Cas9 system
Source: Parasit Vectors. 2026 Apr 13;19:221. doi: 10.1186/s13071-026-07387-0 (PMC13185275; doi:10.1186/s13071-026-07387-0)
Supplement: Supplementary file 5 — Additional file 5. Table S5. Shared differentially expressed GRA and MIC genes in both RHΔrop64 and RHΔrop65 knockout strains. [file 13071_2026_7387_MOESM5_ESM.pdf]

Additional file 5: Table S5 Shared differentially expressed GRA and MIC genes in both RHΔ*rop64* and RHΔ*rop65* knockout strains.

Gene ID	Gene type	Product description	Regulation
TGME49_202620	GRA	Dense granule protein GRA64	Up
TGME49_217410	GRA	Hypothetical protein	Up
TGME49_217530	GRA	Dense granule protein GRA63	Up
TGME49_222170	GRA	Dense granule protein GRA17	Up
TGME49_232270	GRA	Histidine acid phosphatase superfamily protein	Up
TGME49_247440	GRA	Dense granule protein GRA33	Up
TGME49_260520	GRA	Dense granule protein GRA53	Up
TGME49_268790	GRA	Dense granule protein GRA58	Up
TGME49_270250	GRA	Dense granule protein GRA1	Up
TGME49_286450	GRA	Dense granule protein GRA5	Up
TGME49_308970	GRA	Dense granule protein GRA12D	Up
TGME49_215220	GRA	Dense granule protein GRA22	Down
TGME49_240060	GRA	Inhibitor of STAT1 transcription	Down
TGME49_241240	GRA	Dense granule protein GRA65	Down
TGME49_243200	GRA	Inner membrane complex protein IMC29	Down
TGME49_244280	GRA	Hypothetical protein	Down
TGME49_258870	GRA	Cyst wall protein CST7	Down
TGME49_264660	GRA	SAG-related sequence SRS44	Down
TGME49_273560	GRA	Kinesin heavy chain, putative	Down
TGME49_205680	MIC	Hypothetical protein	Up
TGME49_232400	MIC	PAN domain-containing protein	Up
TGME49_234380	MIC	Microneme protein MIC21	Up
TGME49_260190	MIC	Microneme protein MIC13	Up
TGME49_294330	MIC	Apical membrane antigen AMA4	Up
TGME49_204130	MIC	Perforin-like protein PLP1	Down
